# Systematic single-cell analysis reveals dynamic control of transposable element activity orchestrating the endothelial-to-hematopoietic transition

**DOI:** 10.1186/s12915-024-01939-5

**Published:** 2024-06-27

**Authors:** Cong Feng, Ruxiu Tie, Saige Xin, Yuhao Chen, Sida Li, Yifan Chen, Xiaotian Hu, Yincong Zhou, Yongjing Liu, Yueming Hu, Yanshi Hu, Hang Pan, Zexu Wu, Haoyu Chao, Shilong Zhang, Qingyang Ni, Jinyan Huang, Wenda Luo, He Huang, Ming Chen

**Affiliations:** 1 Department of Bioinformatics, College of Life Sciences, Zhejiang University, Hangzhou, 310058 China; 2 Bioinformatics Center, The First Affiliated Hospital, Zhejiang University School of Medicine, Hangzhou, 310058 China; 3 Bone Marrow Transplantation Center, The First Affiliated Hospital, Zhejiang University School of Medicine, Hangzhou, 310058 China; 4 Liangzhu Laboratory, Zhejiang University Medical Center, Hangzhou, 310058 China; 5 Institute of Hematology, Zhejiang University, Hangzhou, 310058 China; 6 Zhejiang Province Engineering Laboratory for Stem Cell and Immunity Therapy, Hangzhou, 310058 China; 7 Department of Hematology, The Second Clinical Medical College of Shanxi Medical University, Shanxi Medical University, Taiyuan, 030000 China; 8 Department of Hematology-Oncology, Taizhou Hospital of Zhejiang Province, Linhai, 317000 China; 9 Department of Veterinary Medicine, Zhejiang University College of Animal Sciences, Hangzhou, 310058 China

**Keywords:** Endothelial-to-hematopoietic transition, Transposable element, Hematopoietic stem cell, Inflammatory signaling, Cis-regulatory element, Hypoxia

## Abstract

**Background:**

The endothelial-to-hematopoietic transition (EHT) process during definitive hematopoiesis is highly conserved in vertebrates. Stage-specific expression of transposable elements (TEs) has been detected during zebrafish EHT and may promote hematopoietic stem cell (HSC) formation by activating inflammatory signaling. However, little is known about how TEs contribute to the EHT process in human and mouse.

**Results:**

We reconstructed the single-cell EHT trajectories of human and mouse and resolved the dynamic expression patterns of TEs during EHT. Most TEs presented a transient co-upregulation pattern along the conserved EHT trajectories, coinciding with the temporal relaxation of epigenetic silencing systems. TE products can be sensed by multiple pattern recognition receptors, triggering inflammatory signaling to facilitate HSC emergence. Interestingly, we observed that hypoxia-related signals were enriched in cells with higher TE expression. Furthermore, we constructed the hematopoietic cis-regulatory network of accessible TEs and identified potential TE-derived enhancers that may boost the expression of specific EHT marker genes.

**Conclusions:**

Our study provides a systematic vision of how TEs are dynamically controlled to promote the hematopoietic fate decisions through transcriptional and cis-regulatory networks, and pre-train the immunity of nascent HSCs.

**Supplementary Information:**

The online version contains supplementary material available at 10.1186/s12915-024-01939-5.

## Background

HSCs are pluripotent cells that possess the ability of self-renewal and lineage differentiation to maintain lifelong hematopoiesis. In embryos, HSCs arise from endothelial cells (ECs) with hematopoietic potential in the aorta-gonad-mesonephro (AGM) region, as tracked by time-lapse imaging [1–5]. The EHT process is highly conserved across vertebrate embryos [6], which occurs at embryonic day (E) 10.5–11.5 in mouse [7, 8] and at Carnegie stage 13–17 (4–6 weeks) in human [9, 10]. A sub-cluster of arterial endothelial cells (AECs) is found to undergo fate decisions to become hematopoietic endothelial cells (HECs) [11–13]. Furthermore, at least two types of HSC precursors (distinguished by CD45 expression) have been identified in mouse intra-aortic hematopoietic clusters (IAHCs) [14–17]. Single-cell technologies have expanded our understanding of cellular heterogeneity and complex relationships during developmental hematopoiesis [18, 19]. Utilizing single-cell RNA sequencing (scRNA-seq) and single-cell sequencing assay for transposase-accessible chromatin (scATAC-seq), the continuous EHT trajectory and an intermediate cell population proximal to HECs (termed pre-HECs) have been delineated in mouse [20, 21]. Recently, a signature gene set (RUNX1+HOXA9+MLLT3+MECOM+HLF+SPINK2+) was discovered that can distinguish human HSCs from other hematopoietic progenitor cells, providing the first single-cell landscape of HSC development from origination to maturation in the human embryo [22]. However, the comparability of cell types and conservation of marker genes in human and mouse EHT remain to be systematically investigated.

The EHT process is strictly regulated by multiple factors at the transcriptional and epigenetic levels [23, 24]. Transcription factors such as RUNX1, GFI1, and GATA2 play vital roles in HSC development [25–27]. Signaling pathways, including NOTCH, WNT, YAP, and VEGF, are also involved in HSC fate decisions [24]. Additionally, inflammatory signals [28, 29], such as interleukins (IL-1, IL-3, and IL-6) [30, 31], tumor necrosis factors (TNF), and interferon signals (IFN), have been highlighted to regulate HSC emergence [32, 33]. In the innate immune system, pattern recognition receptors (PRRs) such as Toll-like receptors (TLRs), RIG-I-like receptors (RLRs), NOD-like receptors (NLRs), and C-type lectin receptors (CLRs), are key activators of inflammatory responses [34]. Toll-like receptor 4 (TLR4) has been shown to regulate HSC formation by promoting NOTCH activity through MyD88-mediated NF-κB signaling [29]. A recent zebrafish study revealed that RLRs (RIG-I, MDA5, and LGP2) are involved in HSC formation through the activation of downstream inflammatory signaling, such as TNF receptor-associated factors (TRAFs) [35]. Typically, PRRs induce antiviral immune responses by recognizing nucleic acids produced by exogenous pathogens [36, 37]. During HSC formation, the ligands for PRRs are puzzling because the AGM region is supposed to be a sterile niche. However, abundant transposable elements (TEs) may provide endogenous nucleic acids for PRRs [38]. Interestingly, TE expression has been detected during zebrafish EHT and is demonstrated to affect HSC generation through the RLR pathway [35]. Nevertheless, the contributions of TEs to human and mouse EHT remain largely unknown.

TEs consist of retrotransposons and DNA transposons (DNAs). Retrotransposons are the most abundant TEs in human and mouse, whether long interspersed nuclear elements (LINEs), short interspersed nuclear elements (SINEs) or hominid SVAs (SINE-VNTR-Alu), and long terminal repeats (LTRs), which are also known as endogenous retroviruses (ERVs). TE jumping may induce genome instability and diseases [39, 40]. Therefore, various defense systems have evolved to domesticate TEs, such as chromatin modification, small RNA silencing, and post-transcriptional repression [41, 42]. In vertebrates, the Krüppel-associated box zinc finger protein (KRAB-ZFP) is a prominent silencing system that inhibits TEs through interactions with KAP1 to recruit DNA methyltransferases (DNMT), SETDB1, HP1, and the nucleosome remodeling deacetylase (NuRD) complex [41, 43]. The human silencing hub (HUSH) complex, coupled with the ATPase MORC2, deposits H3K9me3 on TEs. Although TEs are usually silenced, they can be activated in a temporary or tight fashion at both transcriptional and epigenetic levels to shape embryonic development [44, 45]. Single-cell studies have shown that TEs exhibit cell type-specific expression during gastrulation and organogenesis and participate in the dynamic regulation of pluripotency reprogramming and lineage differentiation [46–48]. Related evidence also suggests that TEs can contribute to hematopoietic regeneration and fate decisions [49–51].

In this study, we conducted a comprehensive survey of the genomic landscape and potential regulatory functions of TEs in human and mouse. Through a systematic single-cell investigation, we provide new insights into the contribution of TEs to the expression and regulatory landscape of EHT, which may shed light on the role of TEs in the context of stem cell development and other cell type transition systems.

## Results

### Widespread TEs harbor great regulatory potential in human and mouse

**Fig. 1:**
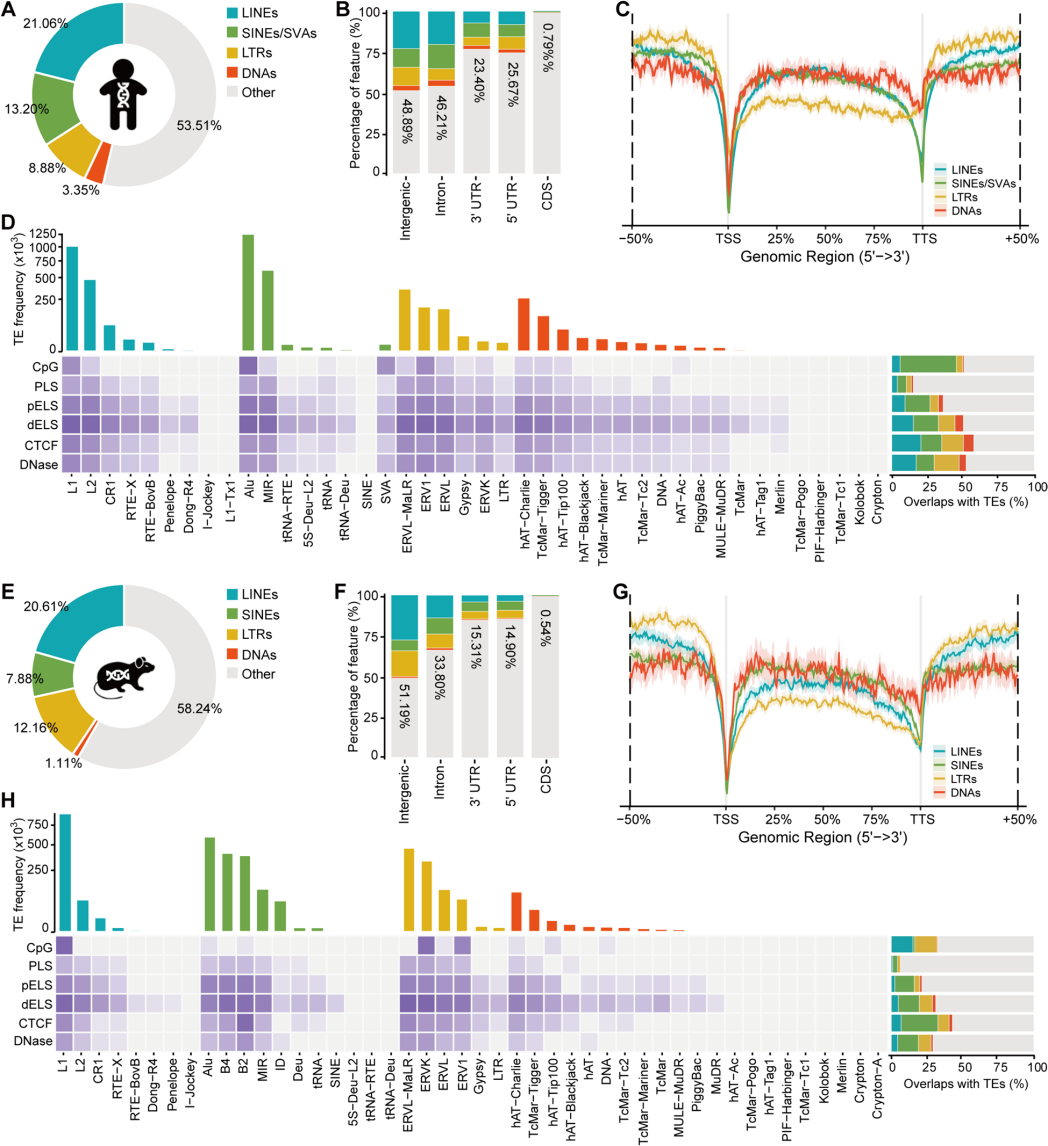
Genomic landscape of TEs in human and mouse. **A**, **E** Genome coverage of TEs in human and mouse. **B**, **F** Overlaps of TEs with gene structures in human and mouse. **C**, **G** Distribution of TEs along the gene body in human and mouse. **D**, **H** The frequency of TE superfamilies (upper bar plot) and overlaps of TEs with CpG islands and cis-regulatory elements (heatmap and right bar plot) in human and mouse

TEs in human and mouse genomes are classified into 4 classes: LINEs, SINEs (including hominid SINE-VNTR-Alu retrotransposons, SVAs), LTRs, and DNAs. These TEs are further categorized into 42 superfamilies (1176 families) and 41 superfamilies (1256 families) in human and mouse, respectively (Additional file 1: Table S1 and S2). TEs constitute approximately 46.38 and 41.76% of the human and mouse genomes (Fig. 1A, E; Additional file 1: Table S3 and S4), with the majority located in non-coding regions, such as intergenic regions, introns, and UTRs (Fig. 1B, F; Additional file 1: Table S5 and S6). Notably, TEs are less abundant near transcription start sites (TSS) and transcription termination sites (TTS) (Fig. 1C, G), potentially maintaining gene transcription specificity [52]. To investigate the regulatory potential of TEs, we assessed the overlap between TE superfamilies and CpG islands, as well as candidate cis-regulatory elements (cCREs) obtained from ENCODE-SCREEN [53]. The cCREs annotations include the genomic locations of promoter-like sites (PLS), proximal enhancer-like signatures (pELS), proximal enhancer-like signatures (pELS), distal enhancer-like signatures (dELS), CTCF signatures, and DNase-H3K4me3 signatures derived from ChIP-seq (H3K4me3, H3K27ac, and CTCF) and DNase-seq data. We employed BEDTools [54] to calculate the intersection between TE annotations and regulatory element coordinates. To enhance the annotation accuracy, we considered a TE to have a potential regulatory function if more than 50% of its length overlapped with a cCRE. In human, SINEs/SVAs contribute to more than 39% of CpG islands (Fig. 1D; Additional file 2: Table S1 and S7). While in mouse, SINEs overlap with only 1.47% of CpG islands, and LINEs (especially L1) and LTRs (ERV1 and ERVK) each contribute to more than 15% (Fig. 1H; Additional file 3: Table S1 and S7). Abundant CpG sites typically maintain TEs in a repressed state. Potentially, TEs can be activated and play a role in embryonic development through demethylation processes, such as epigenetic reprogramming [55, 56]. Among cCREs, a considerable proportion (36.39–57.55% in human and 21.34–42.95% in mouse) of pELS, dELS, CTCF, and DNase-H3K4me3 signatures intersect with TEs (Fig. 1D, H; Additional file 2: Table S2-S6; Additional file 3: Table S2-S6), suggesting that TEs may have evolved regulatory potential and may contribute to pluripotency and early embryogenesis at both transcriptional and epigenetic levels [57–61]. In this study, we focus on analyzing TE expression and chromatin accessibility during EHT to elucidate the potential regulatory mechanisms of TEs in HSC formation.

### TEs are detected with low cell type specificity on the conserved EHT trajectories of human and mouse

**Fig. 2:**
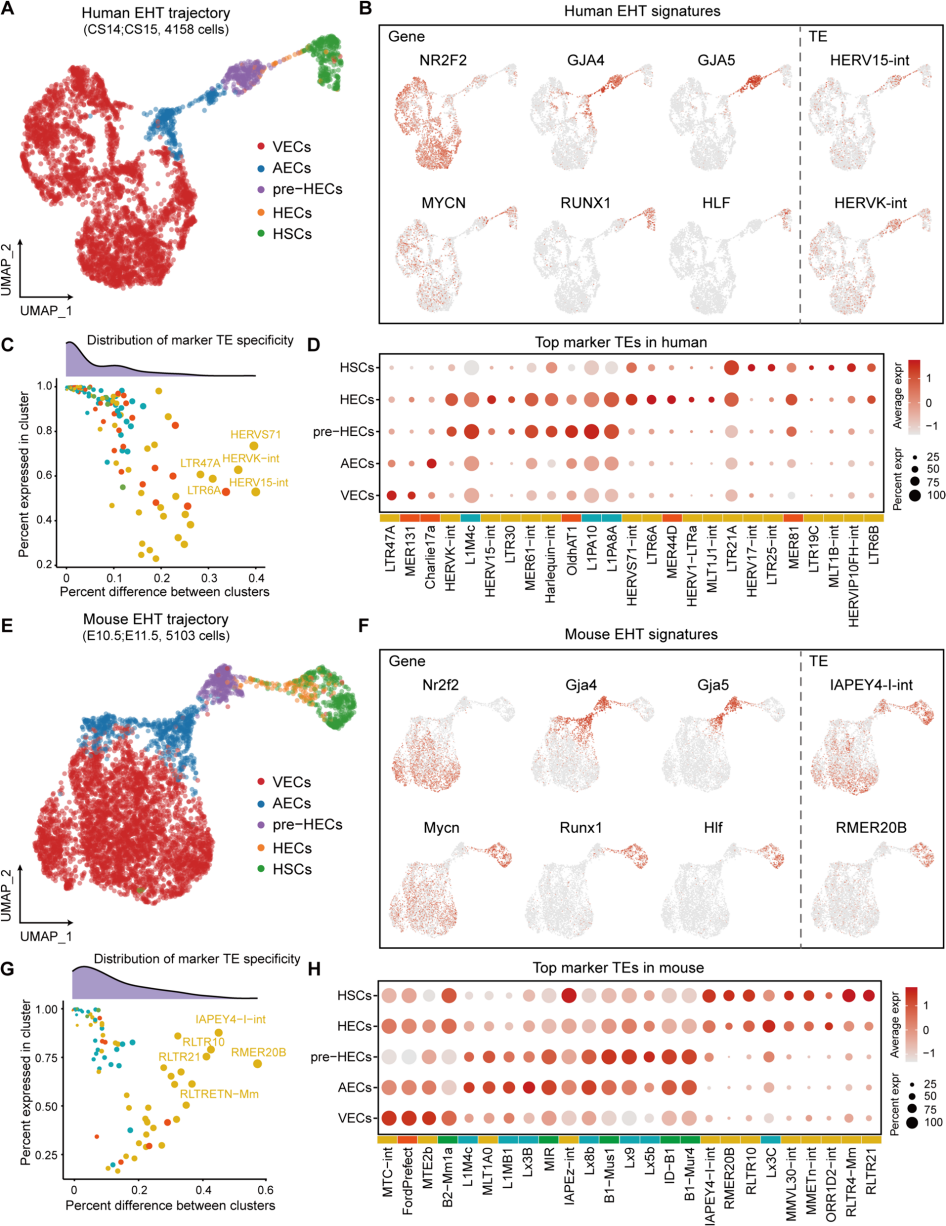
The EHT trajectories and TE expression overview in human and mouse. **A**, **E** Human and mouse EHT trajectories. **B**, **F** Human and mouse EHT signatures. NR2F2 marks VECs, GJA4 marks AECs, GJA5 marks late AECs and pre-HECs, MYCN and RUNX1 marks HECs and HSCs, HLF marks HSCs. These markers are conserved between human and mouse. Only a few marker TEs were identified in the analysis. **C**, **G** The specificities of marker TEs in human and mouse. The majority of TEs with high cell type-specificities are LTRs. **D**, **H** Top markers of each cell type in human and mouse EHT

By using the published scRNA-seq data of human and mouse AGM (Additional file 4: Table S1) [21, 22], we reconstructed the EHT trajectories (Fig. 2A, E ). To characterize the EHT cell types, we identified ECs and HSCs from the AGM UMAP, and annotated cell clusters by using marker genes of VECs (CDH5, NRP2, NR2F2), AECs (GJA4, HEY1, DLL4), pre-HECs (TMEM100, GJA5, EDN1), and HSCs (RUNX1, MYB, HLF) (Fig. 2B, F; Additional file 5: Fig. S1A-C, F and Fig. S2A-C, F). Identification of HECs in UMAP clusters is challenging, and therefore they were annotated based on the signatures of CDH5+ RUNX1+ MYCN+ PTPRC− (Fig. 2B, F; Additional file 5: Fig. S1D-F and Fig. S2D-F). To confirm the consistency of EHT cell types between the two species, we integrated the human and mouse EHT data based on the shared homologous genes. The results showed that the EHT cell types were highly mapped with each other and the majority of EHT markers were conserved (Additional file 5: Fig. S3A, B). For instance, ACE is positive, CD44 is low, and KIT is negative in both human and mouse pre-HECs [20, 62]. However, IL33 and SPINK2 are only expressed in human pre-HECs and HSCs, respectively, while in mouse, Ikzf2 is more enriched in HECs and HSCs (Additional file 5: Fig. S3D).

To explore the TE dynamics on the EHT trajectories, we computed the family-level TE expression using scTE [48]. In scTE, reads mapping to any TE copy in the genome are collapsed to a single TE subtype, reducing errors in multi-mapped read allocation. The differential expression analysis showed that the overall average log2FC values of TEs were relatively low. We selected 0.25, which is close to the median of average log2FC values, as the threshold to obtain relatively more differentially expressed TEs (named marker TEs). Finally, we identified 214 and 96 marker TEs in human and mouse EHT clusters (average log2FC≥0.25 and adjusted *P*-value≤0.05), respectively. Among these, 198 TEs (92.52%) in human were enriched in pre-HECs, while 72 TEs (75%) in mouse belonged to AECs and pre-HECs (Additional file 6: Table S1 and Table S2). Notably, in human EHT, HERV15-int and HERVK-int appeared to be enriched in pre-HECs and HECs, whereas in mouse EHT, IAPEY4-I-int and RMER20B were highly expressed in HSCs (Fig. 2B, F). We calculated marker TE specificity as the difference between the percentage expression of a TE in a cluster and its percentage expression in the remaining clusters. In cell types where TEs were expressed, most of them show low cell type specificities (Fig. 2C, G). The marker TEs that showed relatively higher specificities (percent difference>25%) are mostly LTRs or ERVs, which is consistent in both human and mouse. The top marker TEs for each cell type of human and mouse EHT were displayed in Fig. 2D, H.

### TEs form a distinguished co-upregulation pattern during human pre-HEC and mouse AEC/pre-HEC specification

**Fig. 3:**
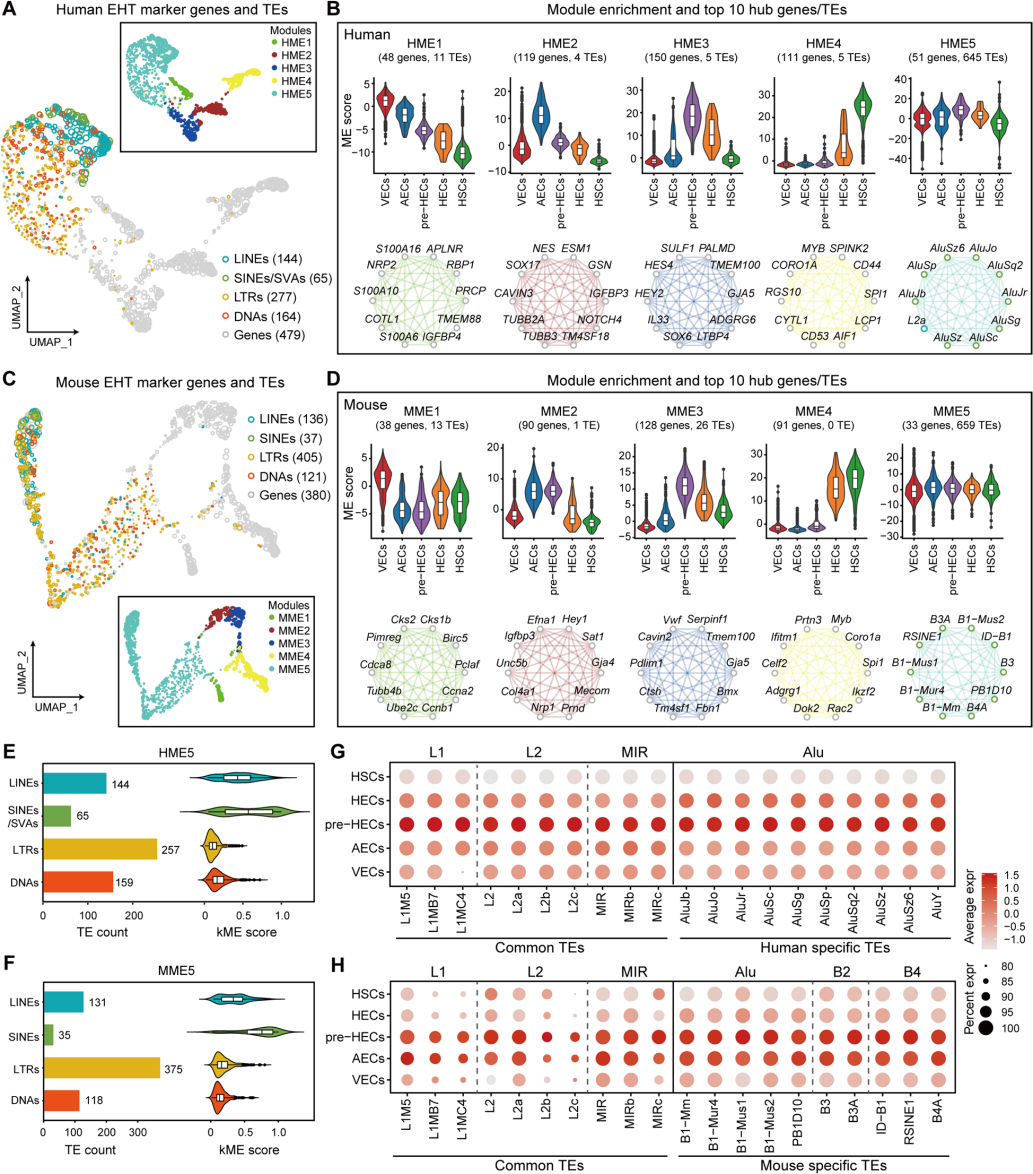
Co-expression network analysis of marker genes and expressed TEs. **A**, **C** Co-expression modules of marker genes and TEs in human and mouse. Most TEs tend to cluster together as distinct modules (HME5 and MME5). **B**, **D** The expression patterns and top 10 hub genes/TEs of each module in human and mouse. TEs show a common upregulation trend in pre-HECs. In the case of mouse, this upregulation appears to occur even earlier, during the AEC stage. **E**, **F** TE composition (bar plot) and module connectivity (kME, violin plot) of HME5 and MME5. **G**, **H** Dot plots show the expression levels of selected common and species-specific TEs

To identify modules that may participate in common regulatory processes during EHT, we performed co-expression network analysis using hdWGCNA [63]. This approach allows us to identify groups of genes and transposable elements (TEs) that exhibit similar expression patterns across different cell types during EHT. A total of 988 filtered TEs, 528 marker genes (average log2FC≥0.5 and adjusted *P*-value≤0.05) in human and 864 filtered TEs, 421 marker genes in mouse were included for co-expression analysis (Additional file 6: Table S3 and S4). Those selected genes and TEs were clustered into 5 modules (HME1-5 and MME1-5) in both human and mouse, in which HME1-4 (MME1-4) were enriched in VECs, AECs, pre-HECs, and HECs/HSCs, respectively (Fig. 3A–D; Additional file 7: Table S1 and S2). The top 10 hub genes/TEs of each module are listed in Fig. 3B and D. Conserved hub genes, such as GJA5 and TMEM100 in pre-HECs, and MYB, SPI1, and CORO1A in HSCs, were found in both human and mouse. Notably, most of the filtered TEs (645 of 670 in human and 659 of 699 in mouse) were clustered in HME5 and MME5 (Fig. 3B, D). These TEs were expressed throughout the entire EHT peaking in human pre-HECs. In mouse samples, TEs were significantly upregulated at the AEC stage, continuing into the pre-HEC stage. Although LTRs accounted for the largest proportion of TEs in HME5 and MME5 (39.84 and 56.90% in human and mouse, respectively), their module connectivity scores (kME scores) were relatively low (Fig. 3E, F). The kME score, defined as the correlation between the expression profile of a feature and the module in a co-expression network by WGCNA [64], measures how strongly a TE is connected to a particular module. Therefore, the lower kME scores of LTRs indicate that they may not be the hub nodes of the network. In contrast, SINEs and LINEs exhibited higher kME scores in both HME5 and MME5, suggesting their potential role as core regulators in the network. Among TEs with kME scores greater than 0.3, L1 was the most abundant in both human and mouse (94 in 190 and 68 in 179) (Additional file 7: Table S1 and S2). Furthermore, 260 common TEs in human and mouse are found to be enriched in HME5 and MME5 (Additional file 7: Table S3). Some top-ranked (according to kME scores) common TEs (L1, L2, and MIR) and species-specific TEs (Alu and mouse-specific B2 and B4) were displayed in Fig. 3G, H.

### TE silencers are transiently relaxed in human pre-HECs and mouse AECs/pre-HECs

**Fig. 4:**
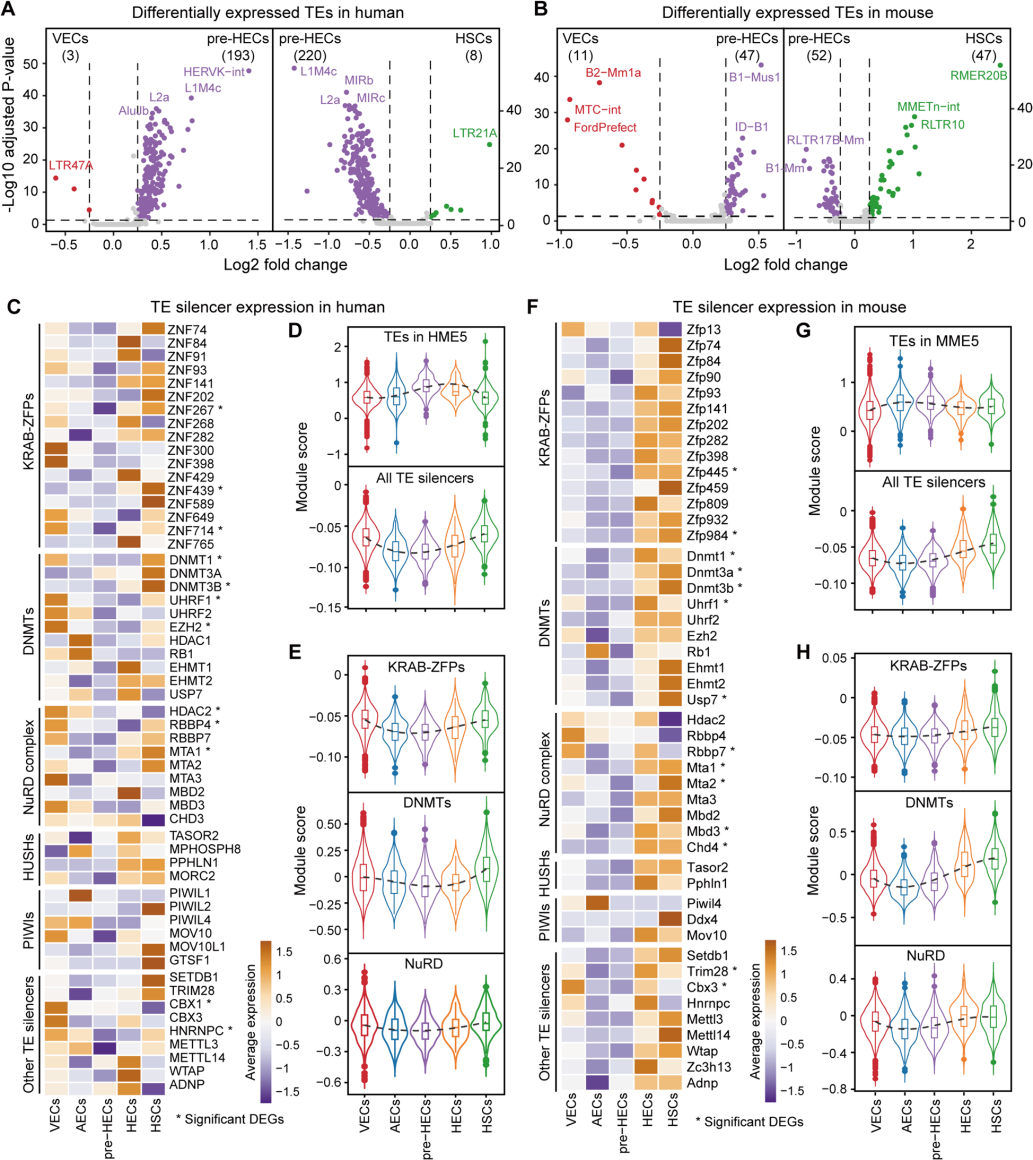
Expression of known TE silencers in human and mouse. **A**, **B** Differential expression of TEs in pre-HECs versus VECs and HSCs in human and mouse. **C**, **F** Expression heatmap of known TE silencing systems in human and mouse, including KRAB-ZFPs, DNMTs, NuRD complex, HUSHs, PIWIs and other TE silencers. **D**, **G** Expression trend of TEs (HME5 and MME5, kME≥0.3) and all TE silencers. **E**, **H** Expression trend of specific TE silencing systems in human and mouse

Differential expression analysis revealed pervasive upregulation of TEs in pre-HECs against VECs and HSCs (Fig. 4A, B; Additional file 8: Table S1-S4). However, in mouse, TE activation was already observed in AECs, possibly due to the presence of more pre-HEC-primed cells (Fig. 3D). As TEs are normally repressed, their transient activation in pre-HECs is likely due to the downregulation of TE silencers. We therefore calculated the relative expression (i.e., module score) of each TE silencing module during EHT using AddModuleScore in Seurat [65]. The majority of TE silencers were downregulated in pre-HECs compared to VECs and HSCs (for example, 84.07 and 77.49% of KRAB-ZFPs in human and mouse, respectively) (Fig. 4C, F; Additional file 8: Table S5 and S6; KRAB-ZFP genes are available from [66]). Among the downregulated KRAB-ZFPs, ZNF84, ZNF382, and ZNF429 were found to bind significantly to L1 superfamily in human [67]. In embryonic stem cells, ZNF91 and ZNF93 can respectively repress SVAs and L1 in human [68], while Zfp932 regulates ERVK in mouse. ZNF268, ZNF300, and ZNF589 were shown to be related to hematopoietic differentiation [69]. Co-factors recruited by KRAB-ZFPs (such as TRIM28, CBX3, and SETDB1) and TE silencers closely related to KRAB-ZFPs (such as DNMTs and NuRD complex), also exhibited relatively low expression levels in pre-HECs (Fig. 4C, E, F and H). Additionally, the HUSH complex (HUSHs), P-element induced Wimpy testis-related genes (PIWIs), and other TE silencers were also expressed relatively lower in pre-HECs than in other cell types, despite overall low expression levels (Fig. 4C, F). These findings suggested that various TE silencers were relaxed by specific mechanisms, leading to transient TE activation during pre-HEC specification (Fig. 4D, G). Interestingly, these TE silencers were upregulated after the pre-HEC stage to re-suppress TE activity, explaining why some members of the DNMT complex (e.g., DNMT1 [70] and EZH2 [71]) and NuRD complex (e.g., HDAC1 and HDAC2 [72]) are required for HSC formation [73].

### TE product sensing facilitates immune activation during HSC orientation

**Fig. 5:**
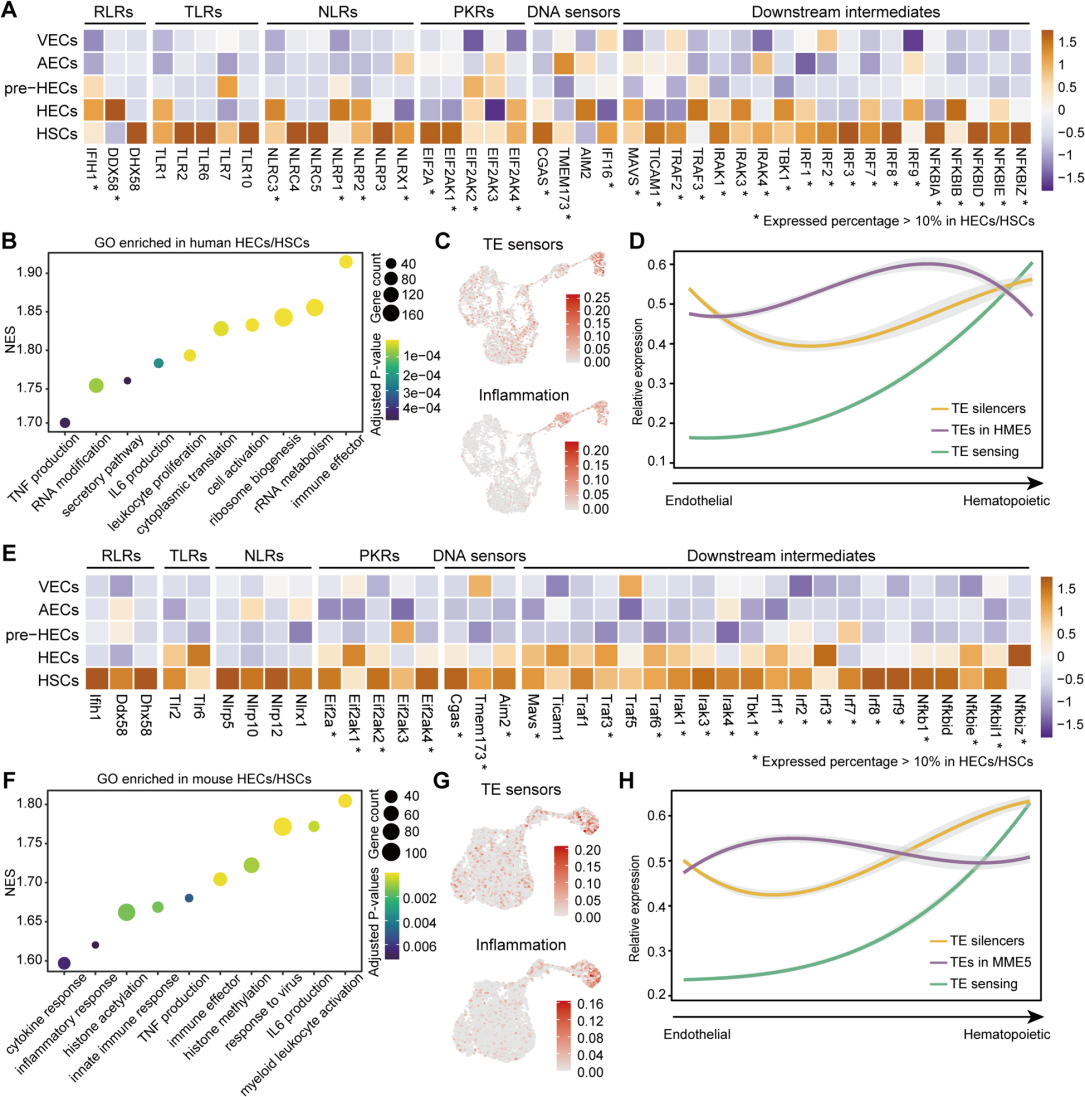
Expression of common TE sensors and functional enrichment for HECs/HSCs. **A**, **E** Expression heatmap of common TE sensors in human and mouse, including RLRs, TLRs, NLRs, PKRs, DNA sensors, and downstream intermediates. **B**, **F** Gene set enrichment analysis of GO terms in human and mouse HECs/HSCs. The NES (normalized enrichment score) represents the degree to which a gene set is overrepresented at the top or bottom of a ranked list of genes. **C**, **G** Module scores of TE sensors and inflammatory genes in human and mouse. **D**, **H** Expression trends of TE silencers, TEs (HME5 and MME5, kME≥0.3), and TE sensing genes during human and mouse EHT. The expression pattern of TEs is opposite to that of TE silencers, whereas TE sensors are less active until the HSC stage

As a main source of endogenous nucleic acids [38, 41], TE products likely activate downstream inflammatory signaling pathways through PRRs. We identified upregulation of a large number of RNA and DNA sensors both in human and mouse HECs/HSCs (Fig. 5A, E; Additional file 9: Table S1 and S2). In human HECs/HSCs, IFIH1 (MDA5) and DDX58 (RIG-I) of RLRs, as well as NLRP1, NLRP2, NLRC3, and NLRX1 of NLRs, were significantly upregulated. Although some TLRs also showed upregulation in HECs/HSCs, their expression levels were low in both species (expression percentage<10%). In addition, protein kinase R genes (PKRs), including EIF2A, EIF2AK1, EIF2AK2, and EIF2AK4, were significantly upregulated in both human and mouse HECs/HSCs. DNA sensors, such as cGAS/STING (TMEM173), were possibly activated by cDNA intermediates of retrotransposons [74, 75]. Typical downstream intermediates of RLRs and cGAS/STING, including MAVS, TRAF3, TBK1, IRF3, and NF-κB, showed highly conserved upregulation patterns (Fig. 5A, E; Additional file 9: Table S3 and S4). Functional enrichment analysis detected both interferon alpha (IFNα) and interferon gamma (IFNγ) response pathways in HECs/HSCs, confirming the activation of these PRRs (Additional file 9: Table S5 and S6). Interestingly, IFNAR1 and IFNGR2 consistently appeared to function earlier in both species (immediately after TE upregulation), while IFNAR2 and IFNGR1 showed complementary patterns (Additional file 9: Table S3 and S4). Gene set enrichment analysis (GSEA) on gene ontology (GO) revealed inflammatory signals (e.g., TNF and IL6) and immune response in HECs/HSCs (Fig. 5B, F; Additional file 9: Table S5 and S6). Taken together, we speculated that TE products (elevated in human pre-HECs or earlier in mouse AECs) could induce inflammatory signals through PRRs during EHT, and trigger immune response pathways to activate HSC progression (Fig. 5C, G and D, H ).

### TE accessibility is dynamically controlled during EHT

**Fig. 6:**
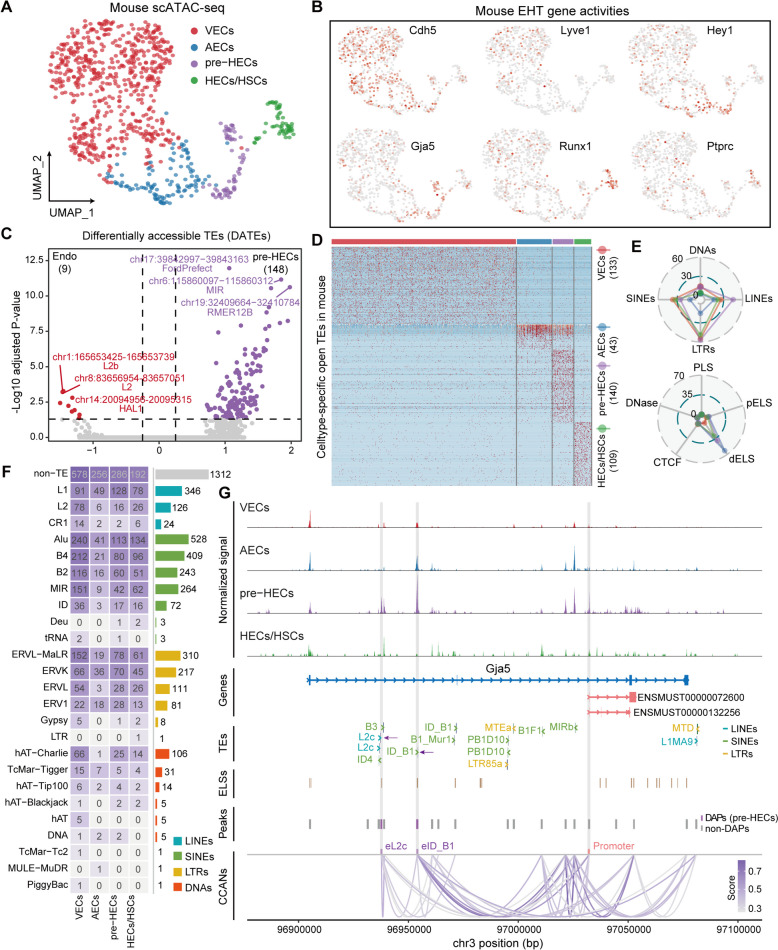
Mouse AGM scATAC-seq analysis and accessible TE identification. **A** Cell types recovered from mouse scATAC-seq data. **B** UMAP of EHT marker gene activities. Cdh5 marks endothelial cells, Lyve1 marks VECs, Hey1 marks AECs, Gja5 marks pre-HECs and partial AECs, Runx1 marks HECs/HSCs, and Ptprc marks HSCs. **C** Differential accessible analysis of TEs in pre-HECs versus endothelial clusters (VECs and AECs). **D** Cell type-specific open TEs identified in mouse EHT. **E** TE composition and potential cis-regulatory functions of cell type-specific open TEs. **F** Overlaps of all cell type-specific accessible peaks with different TE superfamilies. **G** Tracks of normalized signals in each cell type, genes, TEs, ELSs, peaks, and CCANs. Two potential enhancers derived by TEs and the promoter of Gja5 transcripts are highlighted with light grey bars

To explore the potential cis-regulatory function of TEs on HSC origination, we systematically analyzed the scATAC-seq data in the E10.5 mouse AGM. Using cell types transferred from scRNA-seq data, a coherent EHT process was achieved based on scATAC-seq data (Fig. 6A; Additional file 10: Fig. S1A-D). The gene activities of EHT signatures obtained from scRNA-seq were well-fitted to the EHT clusters (Fig. 6B; Additional file 10: Fig. S1E). Next, we calculated the TE activities in each cell (reflecting the degree of TE accessibility) at the locus level. Differential accessibility analysis revealed that TEs were more accessible in pre-HECs compared to endothelial cells (Fig. 6C; Additional file 11: Table S1), aligning with the elevated TE expression in AECs/pre-HECs (Fig. 4A, B). A total of 148 differentially accessible TEs (DATEs, average log2FC≥0. 25 & adjusted *P*-value≤0.05) were enriched in pre-HECs (Fig. 6C), while few DATEs were identified between pre-HECs and HECs/HSCs (Additional file 11: Table S2). Notably, more differentially accessible peaks (DAPs) were found in pre-HECs compared to endothelial cells when analyzing all peaks, suggesting chromatin reprogramming in pre-HECs. Unlike the low specificity of TE expression in scRNA-seq data, a considerable number of cell type-specific open TEs were identified in scATAC-seq data (Fig. 6D; Additional file 11: Table S3). DNAs accounted for the least amount of these cell type-specific open TEs, and LINEs accounted for more in pre-HECs than in other cell types (Fig. 6E). As expected, the majority of open TEs overlapped with the distal enhancers annotated by ENCODE, suggesting their potential enhancer roles.

Differential accessibility analysis for all peaks identified 4230 cell type-specific DAPs, of which 2918 overlapped with TEs, named TEPs (Fig. 6F; Additional file 11: Table S4). The gene regions closest to these TEPs contained many EHT-associated signatures, such as Gja4 (AECs), Gja5, Edn1 (pre-HECs), and Gata2, Cd44, Runx1 (HECs/HSCs) (Additional file 10: Fig. S2; Additional file 11: Table S4). Gja5, a member of the connexin gene family, had elevated expression in mouse AECs and pre-HECs (Fig. 2B, F). The promoter of two Gja5 transcripts (ENSMUST00000072600 and ENSMUST00000132256, annotated in EPD [76]) was specifically more accessible in pre-HECs (Fig. 6G), while two upstream enhancer-like peaks showed high accessibility in both AECs and pre-HECs, potentially permitting earlier Gja5 expression in AECs. Two TEs (chr3:96937220-96937315:L2c and chr3:96954448-96954510:ID_B1) inside the Gja5 gene body may function as enhancers (termed eL2c and eID_B1) to promote Gja5 expression in pre-HECs. These TE regions are also annotated as ELSs in ENCODE. We applied Cicero [77] to predict the cis-co-accessibility networks (CCANs) among peaks detected near or inside Gja5. Cicero calculates a co-access score (representing the strength of co-accessibility) for each pair of peaks, and links with a co-access score lower than 0.4 are filtered to reduce false positives. Although the potential enhancer eID_B1 had the greatest increase in accessibility in pre-HECs, it was also open in AECs and may interact with the two upstream promoter-like regions (Fig. 6G). The potential enhancer eL2c was only opened in pre-HECs, consistent with the accessibility pattern of the proximal promoter, and thus could be more likely to cooperatively increase Gja5 expression. However, the reason for Gja5 upregulation in pre-HECs remains to be further explored, although a recent study indicated its importance for HSCs to dampen oxidative stress [78].

### Cell type-specific accessible TEs shape the hematopoietic *cis*-regulatory networks

**Fig. 7:**
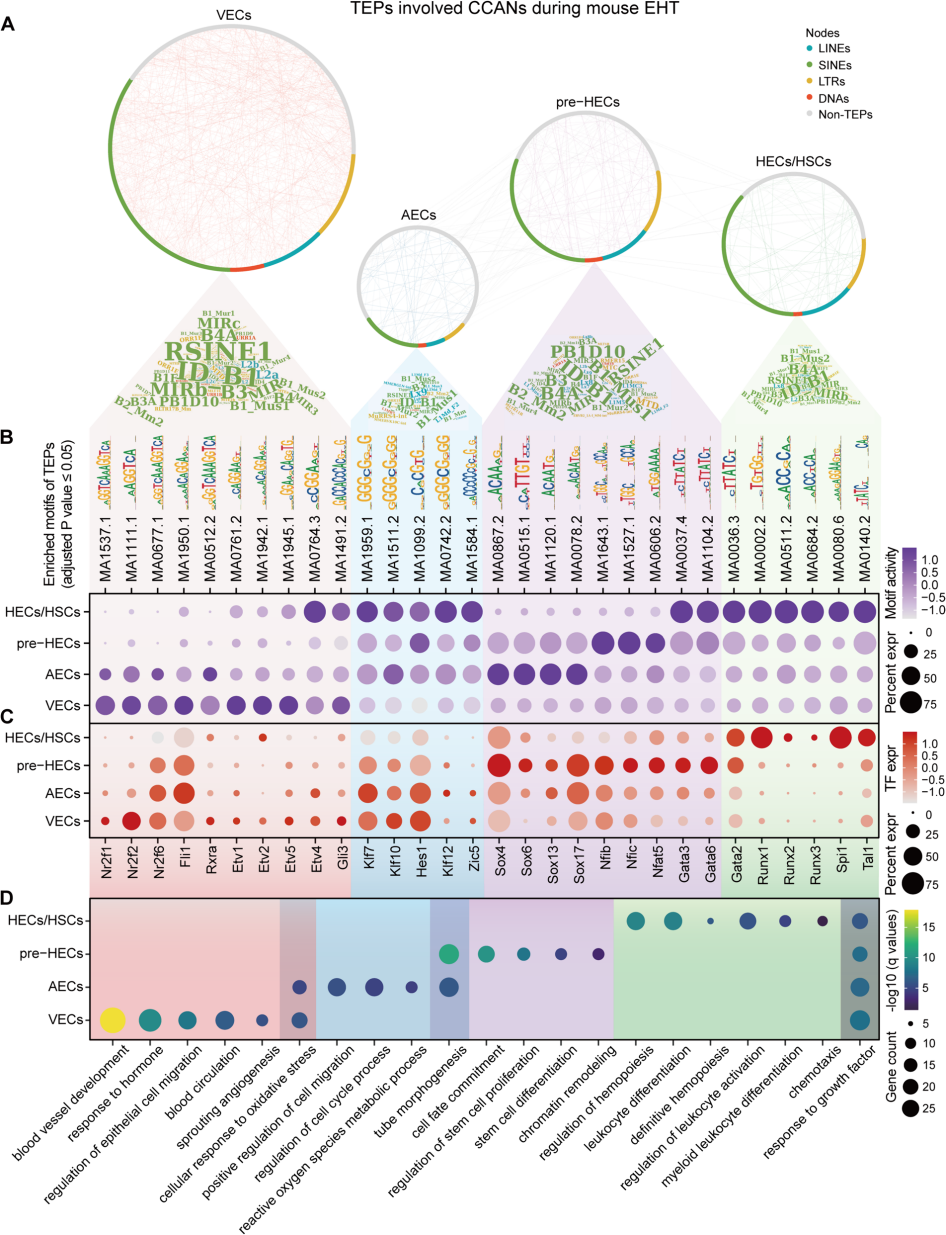
Cis-regulatory network analysis of TE-associated accessible peaks. **A** Cell type CCANs predicted by Cicero. TE families in each cell type are displayed as word clouds. **B** Enriched motifs of TE-associated accessible peaks in each cell type. **C** TF expression corresponding to motifs in **B**. The expression data is extracted from scRNA-seq data. **D** GO enrichment of TF and target genes in each cell type. Overlaps are GO terms common to two or more cell types

While some TEs have been found to act as enhancers driving the expression of hematopoietic-related genes, the co-regulation mode of these TEs remains unclear. To address this, we used Cicero to construct the CCANs of all cell type-specific DAPs (including TEPs and non-TEPs) by filtering out links with a co-access score less than 0.4 (Fig. 7A; Additional file 11: Table S5). Analysis of TE compositions of cell type-specific TEPs revealed that ID_B1 (Alu superfamily) was abundant in all cell types. The top-ranked TEs showed high consistency across cell clusters but were enriched to different motifs in different EHT stages (Fig. 7B; Additional file 11: Table S6), possibly related to the variation accumulated on different TE copies during evolution [79, 80]. Surprisingly, TEs were found to participate in shaping most cis-regulatory networks closely related to the EHT process. For example, TE-involved SOX and GATA binding sites were mostly open in VECs, while TE-involved RUNX binding sites gained increased accessibility in HECs/HSCs. A joint analysis of the enriched motifs and the corresponding transcription factors (TFs) (Fig. 7B, C) revealed that although KLF motifs (Klf7, Klf10 and Klf12) were active in AECs and later stages, these TFs were downregulated to control the developmental fate of AECs. SOX motifs significantly increased activity in AECs in advance, but the expression of TFs (Sox4, Sox6, Sox13, and Sox17) peaked after entering the pre-HEC stage. These motif regions may be cooperatively bound by other TFs in addition to the SOX family, as supported by a recent study showing NF-κB collaborating with IRF3 and other factors to promote nucleosome remodeling [81]. This suggests that when a specific TF is not expressed, its potential collaborators make it possible that the motif occupancy can still be detected. Likewise, Gata3 and Gata6 had higher motif activities in both pre-HECs and HECs/HSCs but were only highly expressed in pre-HECs. This dual regulation via motif binding activity and TF expression precisely shapes the lineage determination during EHT.

Prediction of cell type-specific TF-target networks based on interactions from TRRUST [82] revealed that some EHT signatures were involved downstream of TE-bound TFs (Additional file 10: Fig. S3; Additional file 11: Table S7), such as Kdr, Flt1 (VECs), Smad6, Vegfc (pre-HECs) and Kit, Ikzf1 (HECs/HSCs). GO enrichment analysis showed that these cis-regulatory networks shaped by cell type-specific TEs were enriched in various known functional modules during EHT (Fig. 7D; Additional file 11: Table S8).

### The hypoxic AGM niche may be partially responsible for the transient TE activation preceding hematopoietic fate commitment

**Fig. 8:**
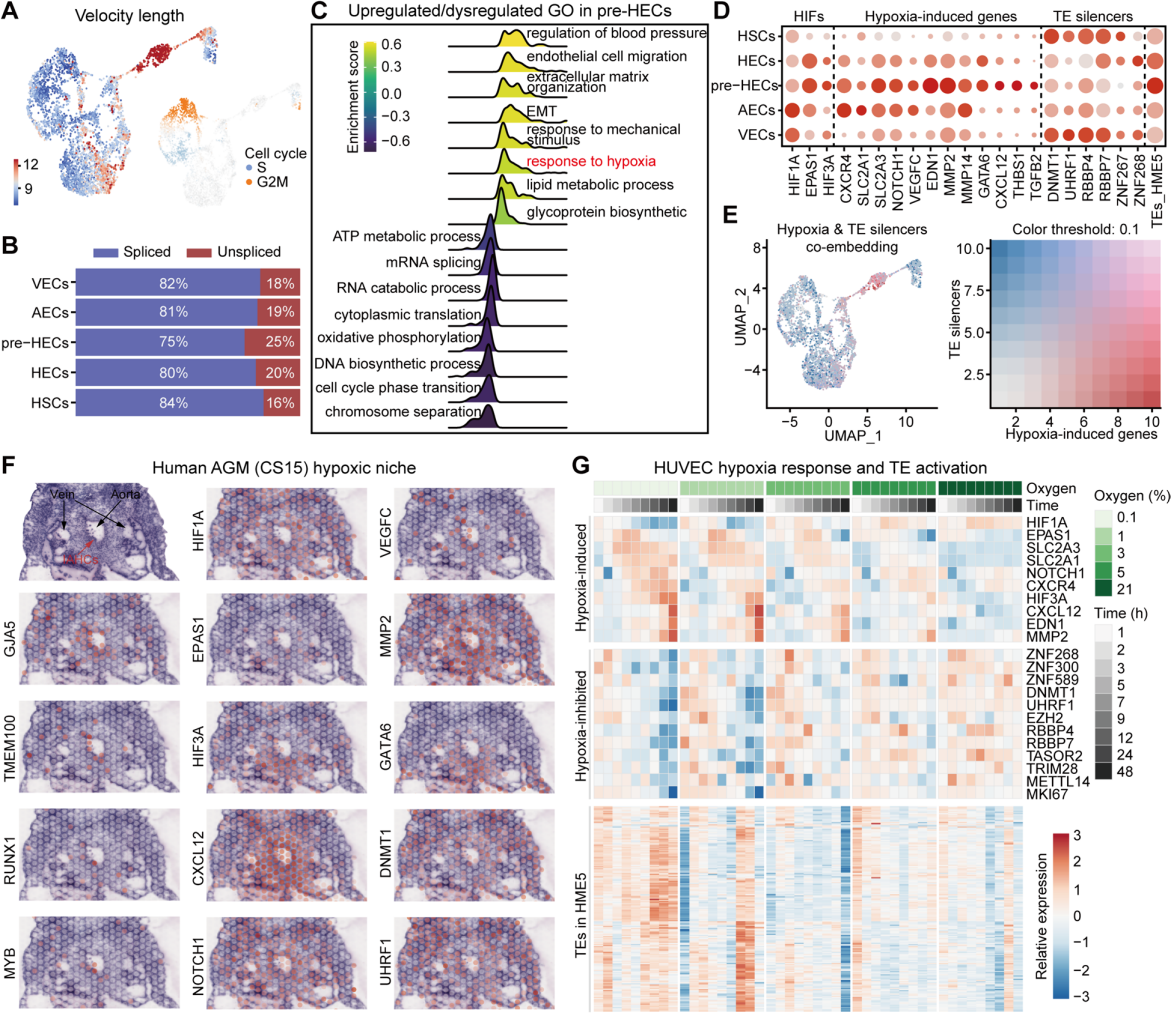
Hypoxia state analysis of pre-HECs and the AGM niche. **A** Velocity length and cell cycle scores on the human EHT UMAP. **B** The proportions of spliced and unspliced RNAs in each cell type. **C** Gene set enrichment analysis of GO terms in human pre-HECs. **D** Expression of hypoxia-related genes and TE silencers in human EHT. **E** Co-embedding of expression of hypoxia-related genes and TE silencers. The expression patterns of the two seem to be opposite. **F** Spatial expression of hypoxia-related genes in the human AGM. **G** Expression heatmap of hypoxia-related genes, potential hypoxia-inhibited genes (TE silencers), and TEs in HME5 (kME≥0.3)

The downregulation of TE silencing systems in pre-HECs may be the main reason for the enhanced TE activity; however, the underlying mechanisms regulating these TE silencers remain unclear. The RNA velocity estimated by Velocyto [83] and scVelo [84] showed that more unspliced RNAs were found in pre-HECs, indicating a significantly high differentiation rate and lack of cell cycle activity (Fig. 8A, B). GSEA analysis showed that mRNA splicing, RNA catabolic process, and cell cycle were downregulated in the pre-HEC stage (Fig. 8C; Additional file 12: Table S1). The downregulation of oxidative phosphorylation and upregulation of lipid metabolic process suggested that pre-HECs may undergo metabolic reprogramming [62]. Notably, genes related to epithelial-to-mesenchymal transition (EMT) and response to hypoxia were upregulated in pre-HECs. Hypoxia has been shown to promote zebrafish HSC formation through hypoxia-inducible factors (HIFs; hif-1a and hif-2a) and Notch signaling [85], and to induce HSPC-like cells from human embryonic stem cells (hESCs) in vitro [86]. The IAHC cluster region in the mouse embryo (E10) was directly observed to be hypoxic by staining [87, 88]. Analysis of hypoxia-related genes in human EHT revealed high expression of EPAS1 (HIF2A) and HIF3A [89] in pre-HECs, while HIF1A was more expressed in VECs and AECs (Fig. 8D; Additional file 12: Table S2). Many hypoxia-induced downstream genes were also found to be enriched in pre-HECs, such as SLC2A3 [90], CXCL12/CXCR4 [91], NOTCH1, VEGFC, EDN1, MMP2/MMP14, GATA6, TGFB2, and THBS1. Spatial transcriptome analysis of human embryo (CS15) demonstrated enrichment of these hypoxia-induced genes in IAHCs (Fig. 8F). The expression patterns of hypoxia-induced genes were opposite to those of TE silencers (Fig. 8D, E), especially DNMT1 and UHRF1 (Fig. 8D, F). To investigate the relationship between hypoxia and TE activation, the TE expression landscape was recalculated in the human AGM dataset. The results indicated local hypoxic areas besides pre-HECs, including stromal cells, which also exhibited higher TE expression levels (Additional file 13: Fig. S1A-C). Few cell type-specific TEs were identified in the AGM region (Additional file 13: Fig. SD), consistent with findings during the EHT trajectory (Fig. 2C, G).

Furthermore, we included a comprehensive time-series RNA-seq study [92] examining the hypoxia response of human umbilical vein endothelial cells (HUVECs) to confirm the relationship between hypoxia and TE activation. TEs were found to be broadly activated after 12 h under extremely low oxygen concentrations (0.1 and 1%), while no significant upregulation was observed in groups with oxygen greater than 3% (Fig. 8G; Additional file 12: Table S3). Different TE classes showed similar upregulation patterns under hypoxia (Additional file 13: Fig. S2). Coincidentally, many TE silencers (e.g., KRAB-ZFP members ZNF268, ZNF300, ZNF589) were greatly downregulated after 12 h of hypoxic culture. HIF3A appeared more correlated with TE expression patterns than HIF1A and EPAS1 (Fig. 8G). Collectively, we hypothesized that the hypoxic AGM niche might induce transient TE activation by inhibiting the expression of TE silencers, which is postulated to be critical for the EHT process (Fig. 5E, J). Analysis of pre-HEC-specific markers in HUVEC data revealed that SOX17, HEY1, and HEY2 were not upregulated under hypoxia (Additional file 13: Fig. S2E, Fig. S3), suggesting distinct roles for these genes during pre-HEC specification.

## Discussion

TEs are abundant in eukaryotic genomes and have evolved essential roles in transcriptional and epigenetic regulation [41, 42, 57, 59, 79]. Recent single-cell sequencing technologies have revealed the broad expression and crucial roles of TEs in developing embryos [46–48]. Although TEs are expressed during definitive hematopoiesis and HSC regeneration [35, 49, 50], the mechanisms of TE activation and their cis-regulatory roles during EHT remain to be investigated. In this work, we demonstrate how cells conservatively program the EHT process and drive HSC formation by dynamically regulating the expression and chromatin accessibility of TEs.

Leveraging the single-cell datasets of human and mouse AGM [21, 22], we reconstructed the EHT trajectories and presented the dynamic landscape of TE expression. Consistent with findings in zebrafish [35], only a few cell type-specific TEs (mainly LTRs) were identified during EHT (Fig. 2C, G). Among marker TEs in human, primate-restricted HERVK transcripts and HERVS71 have been reported to be abundant during human gastrulation [47]. We unexpectedly observed that many TEs were consistently upregulated during human pre-HEC and mouse AEC/pre-HEC specification (Fig. 3B, D; Fig. 4A, B). Coincidentally, TE silencing systems were at relatively low levels from AECs to pre-HECs (Fig. 4C–H), which could partially account for TE activation in this period. Interestingly, two RNA transferases METTL3 and METTL14, which can form nuclear complexes and control TE activity through m6A modification [93, 94], were also downregulated in pre-HECs.

**Fig. 9:**
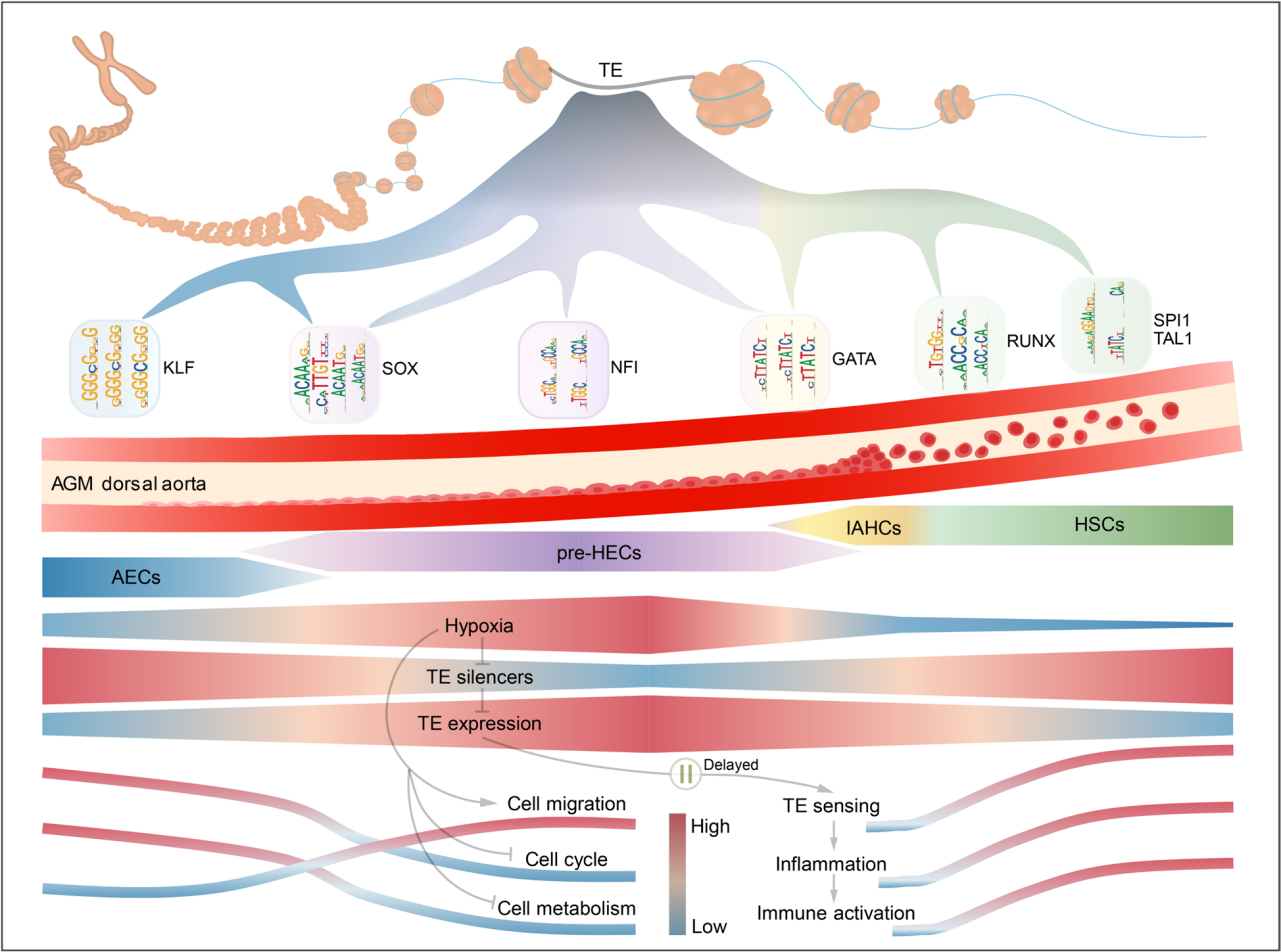
Schematic diagram of dynamic regulations of TEs during EHT. The EHT process in the AGM dorsal aorta is shown in the middle vessel. The process of TEs driving EHT by providing different cis-regulatory elements through dynamic accessibility is presented above. Below the blood vessel, the AGM hypoxic niche induces TE activation in pre-HECs and triggers delayed TE sensing and inflammatory signaling through pattern recognition receptors, thereby promoting the formation of nascent HSCs

Screening common PRRs revealed that many PRRs were upregulated in HECs/HSCs (Fig. 5A, E), suggesting their activation by TE products or other non-coding RNAs [35, 38]. Notably, TE activation and TE sensing are not synchronized, with TEs largely transcribed in human pre-HECs and mouse AECs/pre-HECs not activating PRRs and inflammatory signals until HECs/HSCs. A possible explanation might be that the RNA catabolic process and cell metabolism are quiescent in pre-HECs (Fig. 8B, C), which delays the TE sensing (Fig. 9). When cells are transdifferentiated from pre-HECs into HECs/HSCs, energy metabolism is reactivated, allowing them to respond to various TE products through PRRs. This conserved TE activation and sensing resembles a rehearsal mechanism during the EHT process, allowing nascent HSCs to learn antigen-like properties from endogenous nucleic acid repertoire (e.g., TEs) and complete a pluripotent immune activation.

TEs also exert genome regulatory functions through cis-regulatory elements, especially enhancers [57, 59, 61, 79, 80]. We observed cell type-specific dynamic accessible patterns of TEs (Fig. 6D) and identified two TE-associated enhancers, eL2c and eID_B1, with increased accessibility in pre-HECs and predicted interaction with the Gja5 promoter (Fig. 6G). Through motif prediction and TF expression analysis of cell type-specific TEPs, we recovered the TE-involved cis-regulatory networks during EHT (Fig. 7).

Evidence shows that environmental stress (including heat shock, oxidative, and chemotherapy) [49, 95] is one of the driving forces to induce TE activation. In our study, we inferred that the AGM hypoxic niche may be partially responsible for the activation of TEs in pre-HECs. Hypoxia in the AGM region (IAHC cluster) has already been observed and shown to promote HSC formation [85–88], and our study attributes this role in part to TE activation. Interestingly, stromal and epithelial cells in AGM also exhibited hypoxia and relatively high TE expression (Fig. 8F; Additional file 13: Fig. S1A), indicating that TE activation by hypoxia appears to be cell type insensitive. HIF3A seems to be more related to the TE expression, as evidenced by the upregulation of TEs in stromal and epithelial cells that do not express EPAS1 but express HIF3A (Additional file 13: Fig. S1B, E).

## Conclusions

TEs are domesticated during the evolution of eukaryotic genomes and mediate the emergence of novel regulatory elements [41]. Our study extends the understanding of the potential upstream and downstream effects of TE transcription during EHT at the single-cell level and fills the gap in knowledge of TEs as cis-regulatory elements driving HSC development. Many TEs were found to be upregulated during human pre-HEC and mouse AEC/pre-HEC specification, coinciding with the downregulation of TE silencers. PRR-mediated TE product sensing and activation of inflammatory signaling were delayed until the HSC stage, which may be due to the metabolic reprogramming in pre-HECs. Analysis of scATAC-seq data revealed that dynamically accessible TEs shape the hematopoietic cis-regulatory network to coordinate the EHT process. We additionally reported that the hypoxic AGM niche may be partially responsible for the transient TE activation before hematopoietic fate commitment. Further investigations are required to confirm such a hypothesis. In summary, this study provides a systematical single-cell analysis to uncover how TEs, through dynamic expression and chromatin accessibility, orchestrate the EHT process and drive HSC formation. Our findings contribute to a better understanding of the regulatory roles of TEs in developmental hematopoiesis and provide a foundation for future research in this field.

## Methods

### TE coverage, distribution, and regulatory potential analysis

TE annotations, genomic annotations (intergenic, intron, 3′ UTR, 5′ UTR, CDS), and unmasked CpG islands of human (hg38) and mouse (mm10) were obtained from the UCSC Genome Browser database [96]. The intersection of TEs and gene structures was measured using BEDTools (v2.30.0) [54]. ChIPseeker (v1.34.1) [97] was used to visualize the TE distributions. The reference annotations of cCREs (including PLS, pELS, dELS, CTCF-only, and DNase-H3K4me3) were downloaded from ENCODE [53]. To enhance the annotation accuracy of TE regulatory potential, the overlapping between TE and cCRE should exceed 50% of the TE’s length. The heatmap representations were generated using the R package pheatmap (v1.0.12).

### Single-cell RNA-seq data processing

Raw sequencing data of human and mouse AGM were downloaded from GEO with accession numbers GSE162950 [22] and GSE137117 [21]. The detailed information on samples used in this study can be found in Additional file 4: Table S1. Reads were mapped to the human (refdata-gex-GRCh38-2020-A) and mouse (refdata-gex-mm10-2020-A) reference genomes using CellRanger (v7.1.0). Seurat (v4.3.0) [65] was used to perform downstream analysis. Cells with less than 200 unique molecular identifiers (UMIs) or greater than 15% mitochondrial expression were removed and clusters with unusually low RNA features or counts were also filtered. SCTransform (v0.3.5) [98] was used to normalize the clean data. Batch effects were corrected by Harmony (v0.1.1) [99]. Marker genes were identified using FindAllMarkers with MAST [100]. Cell types were annotated according to the marker genes provided in [22] (Additional file 4). Integration of the human and mouse EHT data was achieved by Seurat CCA based on the shared homologous genes. The R package biomaRt (v2.54.1) [101] was used to map gene symbols of mouse to human.

### Single-cell TE quantification and differential expression analysis

We applied scTE (v1.0) [48] to quantify the TE expression at the family level. To keep the consistency of the read counting results, we incorporated the same gene annotations as CellRanger and TE annotations from UCSC to build the genome indices. The count matrix of only LINEs, SINEs/SVAs, LTRs, and DNAs was kept and merged into the Seurat object. Cell type-specific marker TEs were identified using FindAllMarkers. Differential expression analysis of TEs was performed using FindMarkers.

### Co-expression gene and TE module analysis

Cell type-specific marker genes (average log2FC≥0.5 and adjusted *P*-value≤0.05) and TEs counting more than 50 were extracted for co-expression analysis using hdWGCNA (v0.2.16) [63, 102], which extends the standard WGCNA [64] pipeline into scRNA-seq analysis. Genes and TEs with low expression (less than 50 cells with expression >0) across cells were filtered out. The single cells were first aggregated into pseudobulk (meta) cells to reduce the dropout effect. A similarity matrix was built by calculating the pairwise Pearson correlations between genes and TEs. The similarity matrix was transformed into an adjacency matrix by applying a soft-thresholding power to emphasize strong correlations. Hierarchical clustering was performed to identify modules of genes and TEs with similar expression patterns. The co-expression networks were visualized with UMAP. The kME score was computed based on module eigengene to measure how strongly a gene or TE is connected to a particular module within the network. The module scores of TEs with kME≥0.3 in HME5 and MME5 were calculated using AddModuleScore in Seurat.

### TE silencing and sensing analysis

Genes related to TE silencing were collected from the literature [41, 43]. Potential KRAB-ZFP genes in human and mouse were obtained from [66]. The whole list of TE silencers analyzed in this study can be found in Additional file 8: Table S5 and S6. Genes associated with TE sensing (including PRRs and downstream intermediates) were extracted from publications [34, 36, 38, 74, 75]. TE sensing genes and inflammatory factors are listed in Additional file 9: Table S3 and S4. Differential expression analysis of genes between hematopoietic cells (HECs/HSCs) and endothelial cells (VECs/AECs) was performed using FindMarkers.

### Functional enrichment analysis

Functional enrichment analysis was performed using clusterProfiler (v4.6.2) [103]. GO (biological process) and Molecular Signatures Database (hallmark gene sets) are included.

### Single-cell ATAC-seq data processing

Raw sequencing data of mouse AGM (E10.5) was downloaded from GEO with accession GSE137115 [21]. Reads were mapped to the mouse reference genome (refdata-cellranger-arc-mm10-2020-A) using cellranger-atac (v2.1.0). Signac (v1.9.0) [104] was used to perform downstream analysis, including quality control, normalization, dimension reduction, and clustering. After estimating the gene activities, the cell types of scATAC-seq data were annotated through cross-modality integration and label transfer from scRNA-seq data using CCA [98]. The final cell types were corrected according to the gene activities of known EHT markers.

### Single-cell TE accessibility estimation and differential accessible analysis

The count matrix of TEs was estimated using FeatureMatrix in Signac. Cell type-specific open TEs were identified by FindAllMarkers. Differentially accessible peaks and TEs between cell types were identified using FindMarkers. Each of the open TEs was assigned to the closest gene using ClosestFeature. TE-related differentially accessible peaks (Additional file 10: Fig. S2) were plotted on the mouse genome using karyoloteR (v1.24.0) [105].

### CCAN construction

The cis-co-accessible peaks were identified using Cicero (v1.3.9) [77]. The links with the co-access score of more than 0.4 were extracted to construct the CCAN network, which was visualized in Cytoscape (v3.9.0) [106].

### Motif enrichment and TF expression analysis

The motif enrichment analysis was performed in Signac. The motif position frequency matrices were from JASPAR [107]. Motifs enriched in TE-related differentially accessible peaks were found by FindMotifs. The motif activity was computed by chromVAR (v1.20.2) [108]. Active motifs were selected according to the expression of corresponding TFs from scRNA-seq data. The cell type-specific TF-target network (Additional file 10: Fig. S3) was constructed based on interactions from TRRUST [82]. The average expression of the target genes in the target cell type was required to be more than 0.25.

### Spatial transcriptome data processing

Raw sequencing data of human AGM (CS15, sample 7) spatial transcriptome was downloaded from GEO with accession GSE162950 [22]. Reads were mapped to the human reference genome (refdata-gex-GRCh38-2020-A) using Space Ranger (v2.0.1).

### HUVEC bulk RNA-seq data processing

Raw sequencing data of HUVECs against hypoxia was downloaded from SRA with accession PRJNA561635 [92]. The detailed information on samples used in this study can be found in Additional file 4: Table S2. We treated each sample as a single cell and thus can still use scTE to quantify TE and gene expression. The gene modules of pre-HEC markers (Additional file 13: Fig. S3) in HUVEC data were predicted using WGCNA [64].

### Statistics

Statistical analysis was conducted in R (version 4.2.3). The differential expression testing was achieved with Seurat FindMarkers using Wilcoxon Rank Sum test and *P*-values were adjusted by Bonferroni correction method. GSEA was conducted in clusterProfiler using permutation test and *P*-values were adjusted by Benjamini-Hochberg method.     The scRNA-seq and spatial transcriptome data for human AGM that were analyzed in this study are available from GEO (GSE162950) [22]. The scRNA-seq and scATAC-seq data for mouse AGM are available from GEO (GSE137117) [21]. The time series RNA-seq data for HUVEC are available from SRA (PRJNA561635) [92]. The detailed information on samples used in this study can be found in Additional file 4. All analysis pipelines, in-house scripts and files for reproducing the results in this study can be accessed at GitHub [109] (https://github.com/ventson/hscTE). We also provide a web interface (https://bis.zju.edu.cn/hscTE, implemented using UCSC Cell Browser [110]) to visualize TE and gene expression during human and mouse EHT. The multi-faceted display (including TEs, CpG, cCREs, peaks, and genome coverages) of mouse EHT scATAC-seq data is available from https://bis.zju.edu.cn/hscTE/jbrowse/?data=mouse, which is implemented by JBrowse [111].

### Supplementary Information

Additional file 1: Table S1. TE families, superfamilies and classes in human. Table S2. TE families, superfamilies and classes in mouse. Table S3. Genome coverage of TEs in human. Table S4. Genome coverage of TEs in mouse. Table S5. Genomic distribution of TEs in human. Table S6. Genomic distribution of TEs in mouse.

Additional file 2: Table S1. Overlaps among TEs and CpG islands in human. Table S2. Overlaps among TEs and promoter-like sites (PLS) in human. Table S3. Overlaps among TEs and proximal enhancer-like sites (pELS) in human. Table S4. Overlaps among TEs and distal enhancer-like sites (dELS) in human. Table S5. Overlaps among TEs and CTCF-only sites in human. Table S6. Overlaps among TEs and DNase-H3K4me3 sites in human. Table S7. Copy number of cCREs overlapped with TEs in human.

Additional file 3: Table S1. Overlaps among TEs and CpG islands in mouse. Table S2. Overlaps among TEs and promoter-like sites (PLS) in mouse. Table S3. Overlaps among TEs and proximal enhancer-like sites (pELS) in mouse. Table S4. Overlaps among TEs and distal enhancer-like sites (dELS) in mouse. Table S5. Overlaps among TEs and CTCF-only sites in mouse. Table S6. Overlaps among TEs and DNase-H3K4me3 sites in mouse. Table S7. Copy number of cCREs overlapped with TEs in mouse.

Additional file 4: Table S1. Human and mouse AGM datasets included in this study. Table S2. HUVEC bulk RNA-seq dataset (PRJNA561635) included in this study.

Additional file 5: Figure S1. Steps to reconstruct the human EHT trajectory. Figure S2. Steps to reconstruct the mouse EHT trajectory. Figure S3. Integration of the human and mouse EHT scRNA-seq data.

Additional file 6: Table S1. Marker TEs in human EHT. Table S2. Marker TEs in mouse EHT. Table S3. Marker genes in human EHT. Table S4. Marker genes in mouse EHT.

Additional file 7: Table S1. Co-expressed gene and TE modules in human. Table S2. Co-expressed gene and TE modules in mouse. Table S3. Common TEs in HME5 and MME5.

Additional file 8: Table S1. Differentially expressed TEs between pre-HECs and VECs in human. Table S2. Differentially expressed TEs between pre-HECs and HSCs in human. Table S3. Differentially expressed TEs between pre-HECs and VECs in mouse. Table S4. Differentially expressed TEs between pre-HECs and HSCs in mouse. Table S5. Average expression of TE silencers in human. Table S6. Average expression of TE silencers in mouse.(XLSX 1024 KB)

Additional file 9: Table S1. Differentially expressed genes in human hematopoietic cells (HECs/HSCs) vs endothelial cells (VECs/AECs). Table S2. Differentially expressed genes in mouse hematopoietic cells (HECs/HSCs) vs endothelial cells (VECs/AECs). Table S3. Average expression of pattern recognition receptors and downstream signals in human. Table S4. Average expression of pattern recognition receptors and downstream signals in mouse. Table S5. GSEA analysis of genes in human hematopoietic cells (HECs/HSCs) vs endothelial cells (VECs/AECs). Table S6. GSEA analysis of genes in mouse hematopoietic cells (HECs/HSCs) vs endothelial cells (VECs/AECs).

Additional file 10: Figure S1. Steps to reconstruct the mouse EHT trajectory from scATAC-seq data. Figure S2. Genome landscape of differentially accessible peaks (DAPs). Figure S3. The TF-target network in mouse EHT.

Additional file 11: Table S1. Differentially accessible TEs between pre-HECs and VECs/AECs. Table S2. Differentially accessible TEs between pre-HECs and HECs/HSCs. Table S3. Cell type-specific open TEs. Table S4. Closest genes and annotations of differentially accessible peaks in mouse EHT. Table S5. Co-access scores of cell type-specific peak pairs. Table S6. Enriched motifs of TE associated cell type-specific peaks (TEPs). Table S7. Activated TF-target pairs during EHT (TFs are corresponding to TE-related motifs). Table S8. GO enrichment results of cell type-specific network modules of TE-bound TFs and downstream targets.

Additional file 12: Table S1. GSEA analysis of pre-HECs vs other cell types. Table S2. Average expression of hypoxia-related genes in human EHT cell types. Table S3. Normalized counts of genes and TEs in the HUVEC dataset (PRJNA561635).

Additional file 13: Figure S1. The hypoxic niche and TE expression in human AGM scRNA-seq data. Figure S2. Expression heatmap of each TE family (grouped into four TE classes) in the HUVEC bulk RNA-seq data. Figure S3. Five gene modules of pre-HEC markers on the HUVEC data.

## Data Availability

The scRNA-seq and spatial transcriptome data for human AGM that were analyzed in this study are available from GEO (GSE162950) [22]. The scRNA-seq and scATAC-seq data for mouse AGM are available from GEO (GSE137117) [21]. The time series RNA-seq data for HUVEC are available from SRA (PRJNA561635) [92]. The detailed information on samples used in this study can be found in Additional file 4 . All analysis pipelines, in-house scripts and files for reproducing the results in this study can be accessed at GitHub [109] (https://github.com/ventson/hscTE). We also provide a web interface (https://bis.zju.edu.cn/hscTE , implemented using UCSC Cell Browser [110]) to visualize TE and gene expression during human and mouse EHT. The multi-faceted display (including TEs, CpG, cCREs, peaks, and genome coverages) of mouse EHT scATAC-seq data is available from https://bis.zju.edu.cn/hscTE/jbrowse/?data=mouse , which is implemented by JBrowse [111].
