# Supplementary material for: Systematic single-cell analysis reveals dynamic control of transposable element activity orchestrating the endothelial-to-hematopoietic transition
Source: BMC Biol. 2024 Jun 27;22:143. doi: 10.1186/s12915-024-01939-5 (PMC11209969; doi:10.1186/s12915-024-01939-5)
Supplement: Supplementary file 10 — Additional file 10: Figure S1. Steps to reconstruct the mouse EHT trajectory from scATAC-seq data. Figure S2. Genome landscape of differentially accessible peaks (DAPs). Figure S3. The TF-target network in mouse EHT. [file 12915_2024_1939_MOESM10_ESM.docx]

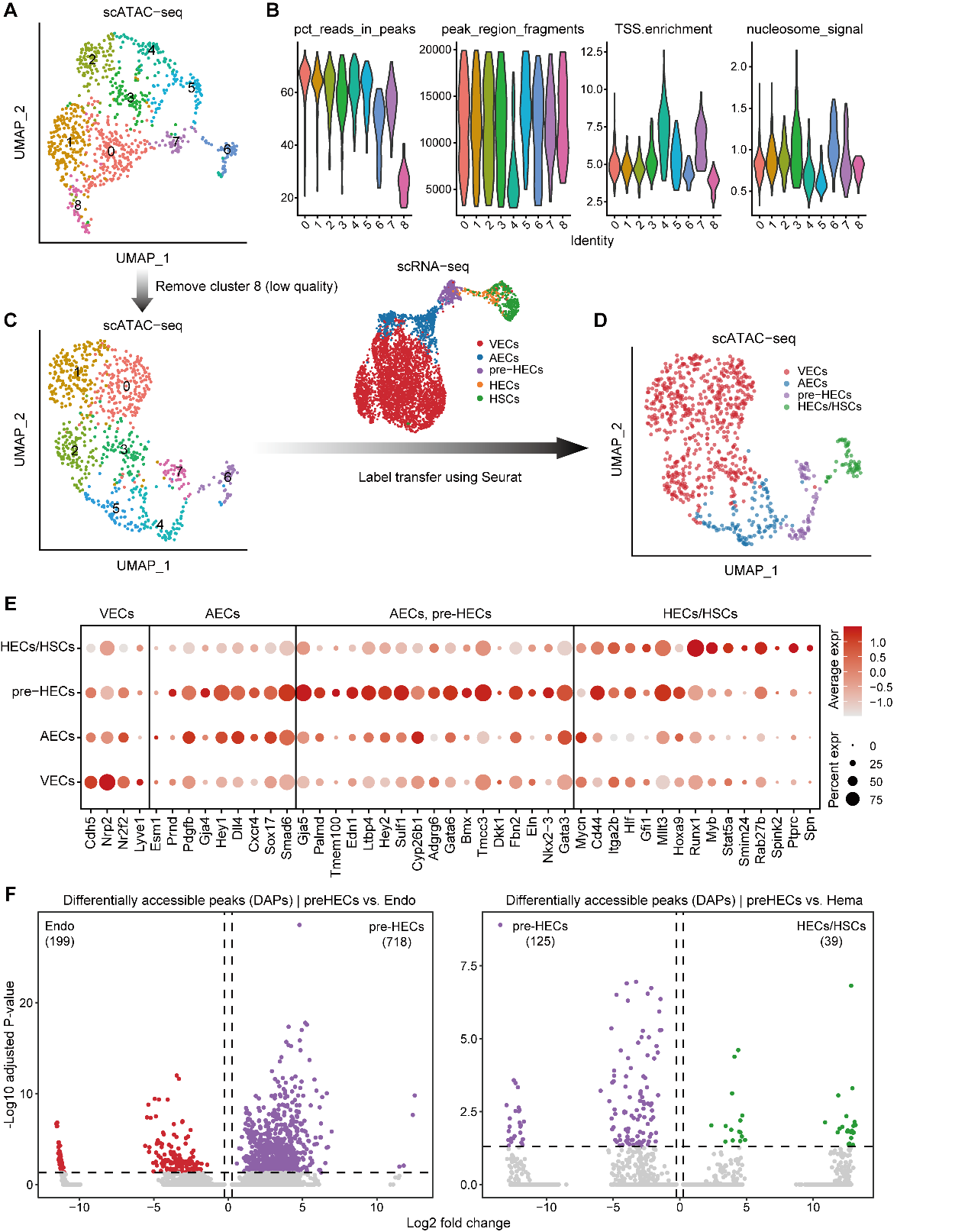


**Figure S1.** Steps to reconstruct the mouse EHT trajectory from scATAC-seq data. **A** The raw UMAP plot of mouse AGM clusters. **B** Quality metrics of mouse scATAC-seq data. **C, D** Filtering and annotating the mouse scATAC-seq data. The cell types are transferred from mouse scRNA-seq data. **E** EHT marker gene activities of mouse EHT cell types in scATAC-seq data. **F** Differential accessible analysis of pre-HECs compared with endothelial cells (VECs/AECs) and hematopoietic cells (HECs/HSCs).


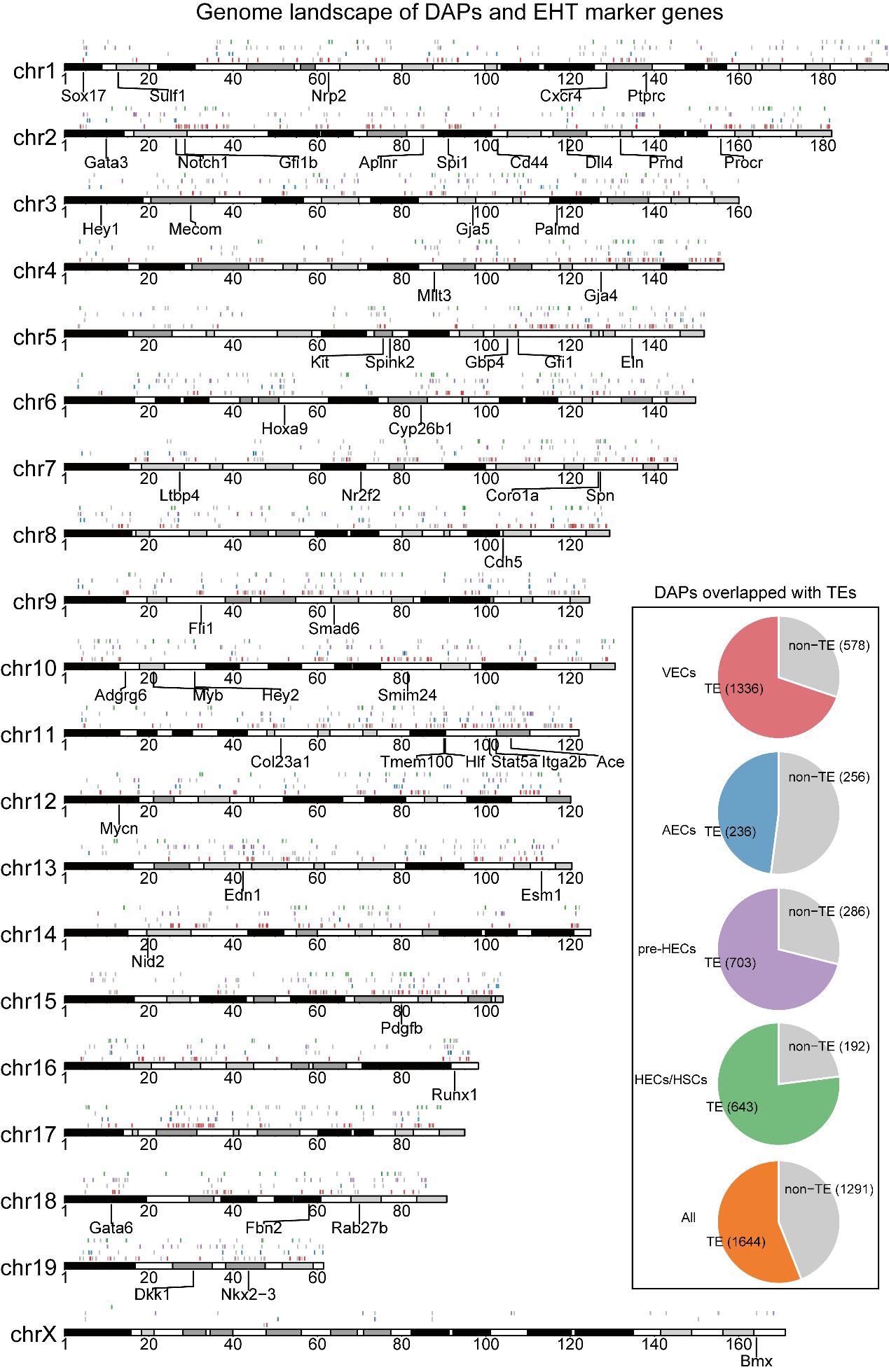


**Figure S2.** Genome landscape of differentially accessible peaks (DAPs). The peaks are grouped into TE and non-TE overlapped. DAPs in different cell types are plotted on four tracks with different colors. Typical EHT marker genes are also indicated on the chromosomes.


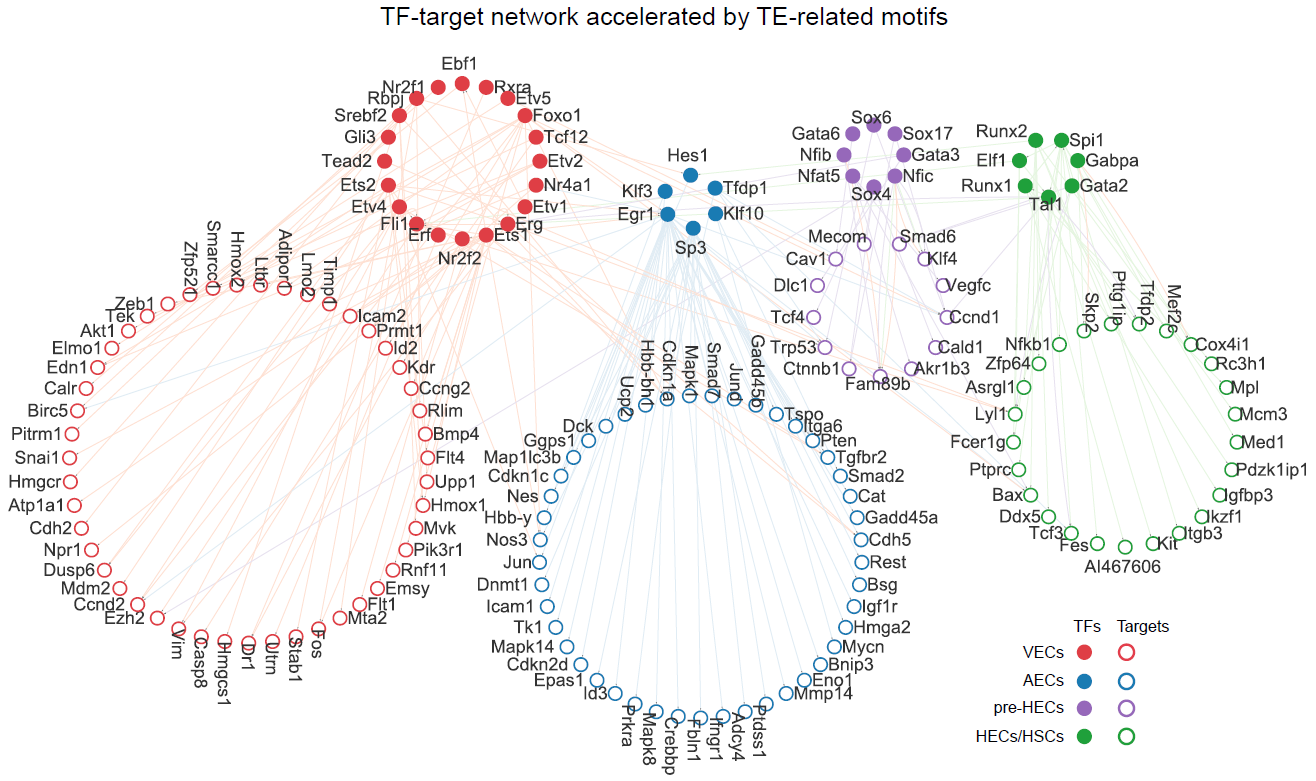


**Figure S3.** The TF-target network in mouse EHT. TFs are selected from TE-related motifs. Targets of TFs are obtained from TRRUST.
