# Supplementary material for: Systematic single-cell analysis reveals dynamic control of transposable element activity orchestrating the endothelial-to-hematopoietic transition
Source: BMC Biol. 2024 Jun 27;22:143. doi: 10.1186/s12915-024-01939-5 (PMC11209969; doi:10.1186/s12915-024-01939-5)
Supplement: Supplementary file 13 — Additional file 13: Figure S1. The hypoxic niche and TE expression in human AGM scRNA-seq data. Figure S2. Expression heatmap of each TE family (grouped into four TE classes) in the HUVEC bulk RNA-seq data. Figure S3. Five gene modules of pre-HEC markers on the HUVEC data. [file 12915_2024_1939_MOESM13_ESM.docx]

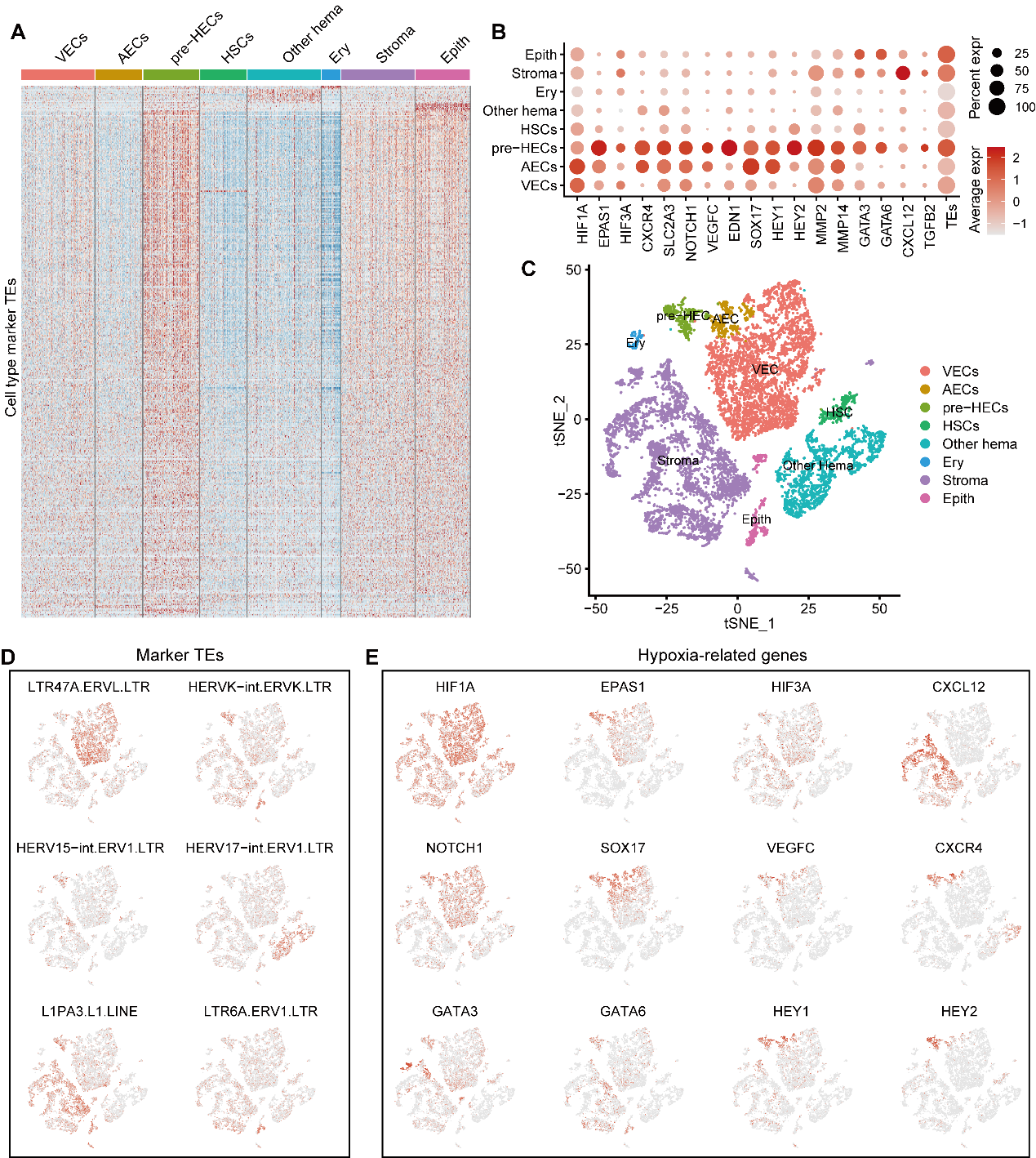


**Figure S1.** The hypoxic niche and TE expression in human AGM scRNA-seq data. **A** Cell type marker TEs in human AGM. In addition to pre-HECs, stromal and epithelial cells also show relatively higher TE expression. **B** Expression of hypoxia-related genes and TE silencers in human AGM. **C** The tSNE plot of human AGM cell types. **D** UMAP of selected marker TE expression in human AGM. **E** UMAP of hypoxia-related gene expression in human AGM.


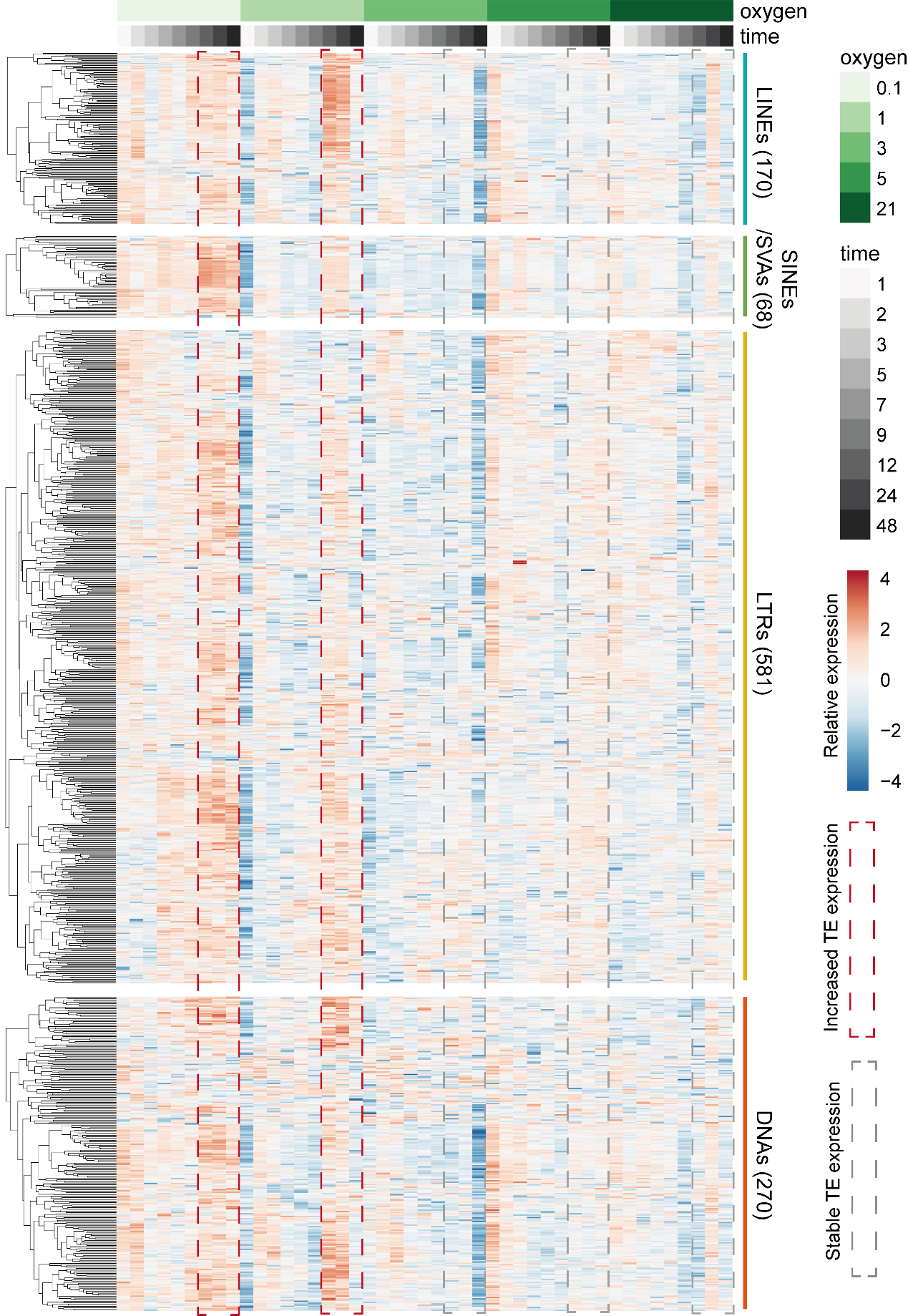


**Figure S2.** Expression heatmap of each TE family (grouped into four TE classes) in HUVEC bulk RNA-seq data.


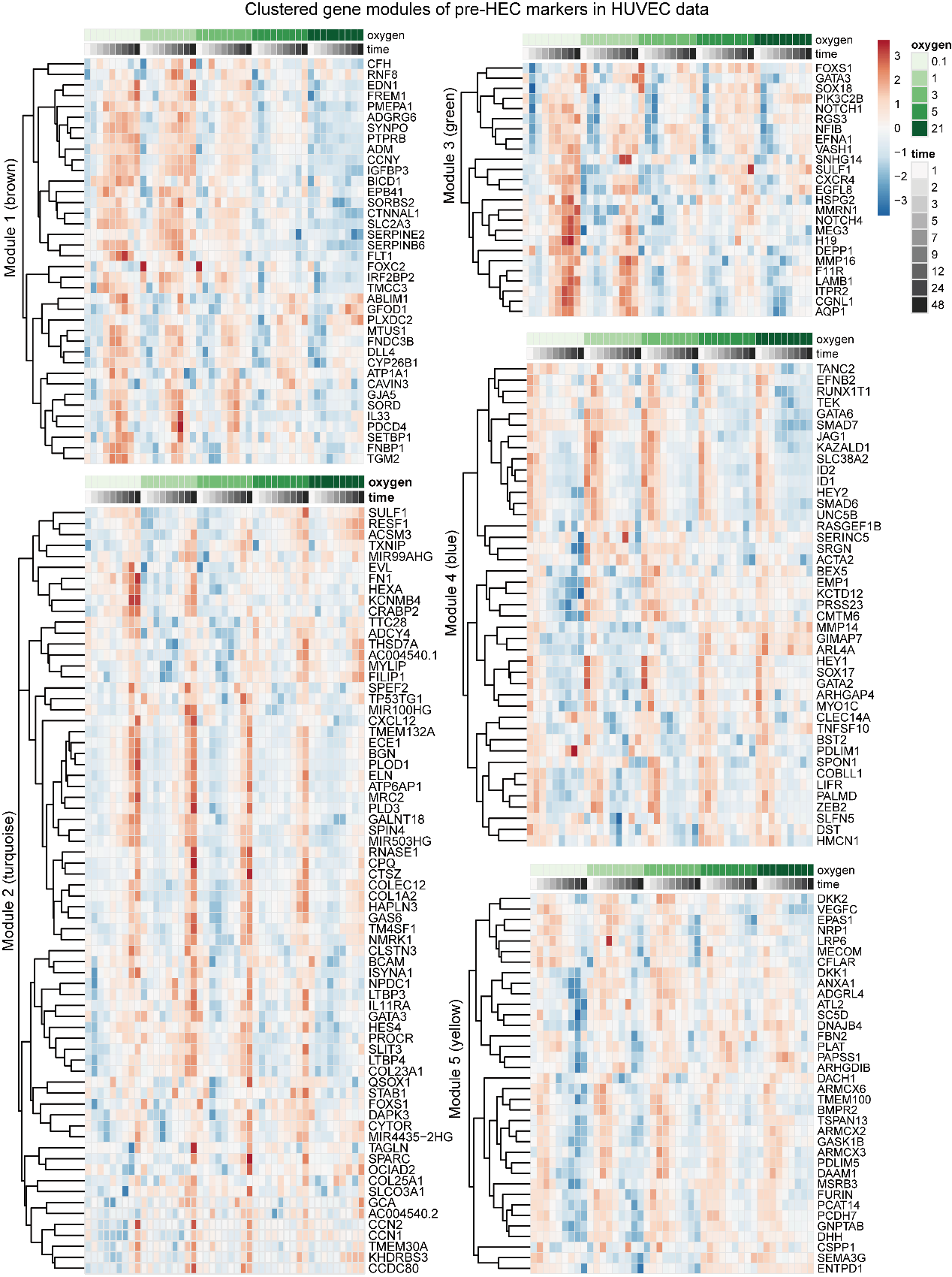


**Figure S3.** Five gene modules of pre-HEC markers on HUVEC data. The gene modules are identified using WGCNA.
